# A Novel NAD-RNA Decapping Pathway Discovered by Synthetic Light-Up NAD-RNAs

**DOI:** 10.3390/biom10040513

**Published:** 2020-03-28

**Authors:** Florian Abele, Katharina Höfer, Patrick Bernhard, Julia Grawenhoff, Maximilian Seidel, André Krause, Sara Kopf, Martin Schröter, Andres Jäschke

**Affiliations:** Institute of Pharmacy and Molecular Biotechnology, Heidelberg University, Im Neuenheimer Feld 364, 69120 Heidelberg, Germany

**Keywords:** Decapping, RNA modification, NAD-capped RNA, glycohydrolase, NAD metabolism, fluorescent RNA, high-throughput screening

## Abstract

The complexity of the transcriptome is governed by the intricate interplay of transcription, RNA processing, translocation, and decay. In eukaryotes, the removal of the 5’-RNA cap is essential for the initiation of RNA degradation. In addition to the canonical 5’-N7-methyl guanosine cap in eukaryotes, the ubiquitous redox cofactor nicotinamide adenine dinucleotide (NAD) was identified as a new 5’-RNA cap structure in prokaryotic and eukaryotic organisms. So far, two classes of NAD-RNA decapping enzymes have been identified, namely Nudix enzymes that liberate nicotinamide mononucleotide (NMN) and DXO-enzymes that remove the entire NAD cap. Herein, we introduce 8-(furan-2-yl)-substituted NAD-capped-RNA (^Fur^NAD-RNA) as a new research tool for the identification and characterization of novel NAD-RNA decapping enzymes. These compounds are found to be suitable for various enzymatic reactions that result in the release of a fluorescence quencher, either nicotinamide (NAM) or nicotinamide mononucleotide (NMN), from the RNA which causes a fluorescence turn-on. ^Fur^NAD-RNAs allow for real-time quantification of decapping activity, parallelization, high-throughput screening and identification of novel decapping enzymes in vitro. Using ^Fur^NAD-RNAs, we discovered that the eukaryotic glycohydrolase CD38 processes NAD-capped RNA in vitro into ADP-ribose-modified-RNA and nicotinamide and therefore might act as a decapping enzyme in vivo. The existence of multiple pathways suggests that the decapping of NAD-RNA is an important and regulated process in eukaryotes.

## 1. Introduction

The selective degradation of individual RNA species is a crucial component in the regulation of gene expression, enabling the cell to respond quickly to changing environmental conditions. In eukaryotes, the removal of the 5’-cap is essential for 5’ to 3’ RNA decay. RNA capping is considered to be a hallmark of eukaryotic gene expression, in which a canonical 5’-N7-methyl guanosine cap (m^7^G) protects mRNA from degradation and modulates maturation, localization, and translation [1].

In 2009, the ubiquitous redox coenzyme nicotinamide adenine dinucleotide (NAD) was discovered as a new RNA modification in bacteria [2], and a few years later we identified the modified RNAs in *Escherichia coli* [3]. NAD was found to be attached to a specific set of regulatory RNAs in bacteria in a cap-like manner, and to modulate the functions of these RNAs [3,4]. To identify NAD-capped RNAs and to enable their functional characterization, we developed a chemo-enzymatic capture approach (NAD captureSeq) [5]. Using this protocol, NAD-RNA conjugates were selectively enriched from *E. coli* total RNA and analysed by next-generation sequencing (NGS). The discovery of NAD-capped RNA [3] in the bacterium *E. coli* provided an unexpected link between redox biology, metabolism, and RNA processing [4], and represented the first description of a prokaryotic cap [6,7]. 

Initially assumed to be prokaryote-specific [3,8,9], this novel RNA cap appears to be conserved in eukaryotic systems, too: NAD-capped RNAs were identified in a human cell line, in the budding yeast *Saccharomyces cerevisiae,* and in the model plant *Arabidopsis thaliana* [10,11,12,13]. The only biosynthesis pathway known to-date is the incorporation of NAD as a non-canonical initiator nucleotide by the cellular RNA polymerases [8,14]. While specific promoter sequences were found to be responsible for efficient NAD incorporation [8,15], the question whether NAD incorporation is a stochastic misincorporation or a regulated event is a matter of debate [16].

Canonical capping and decapping are highly regulated processes, until recently thought to be exclusive for eukaryotes. Multiple decapping enzymes, such as Dcp2 [17] or DXO/Rai1 [18,19], are known to remove the m^7^G cap and thereby trigger different RNA decay pathways [20]. 

The discovery of NAD-capped RNAs in bacteria and eukaryotes laid the foundation for the discovery of decapping enzymes that are able to remove these caps. In *E. coli*, we identified the Nudix NADH-pyrophosphohydrolase NudC as the first bacterial decapping enzyme, which converts 5’-NAD-RNA into 5’-P-RNA, thereby triggering RNase E-mediated RNA decay [3]. In Gram-positive *Bacillus subtilis*, the Nudix hydrolase BsRppH was found to perform the same reaction [8]. Both Nudix hydrolases cleave the phosphoanhydride bond of NAD, thereby releasing nicotinamide mononucleotide (NMN) and 5’-P-RNA, which contains the adenosine of NAD at its 5’-end. Moreover, the mammalian and fungal DXO1/Rai1 enzymes were described to remove—in addition to the eukaryotic m^7^G-cap—the NAD modification. In contrast to the Nudix hydrolases, the DXO enzymes cut off the complete NAD cap including the adenosine, generating a 5’-P-RNA shortened by one nucleotide [10,21]. 

In consideration of the variety of mammalian proteins that show decapping activity on canonically capped RNAs [20], we assumed that there are likely more than the currently known few enzymes that can decap 5’-NAD-RNA. 

The discovery and characterization of decapping enzymes has so far been time-consuming and laborious. Most commonly, ^32^P-radioactively labelled capped RNA substrates [3,22] are added to candidate enzymes, and the reaction mixtures analysed by different types of gel electrophoresis [23,24] or thin layer chromatography (TLC) [22,25]. Alternatively, the decapping mixtures of non-radioactive RNA samples are analysed by liquid chromatography-mass spectrometry (LC-MS) [26,27]. All these techniques are characterized (in addition to specific disadvantages such as the requirement of hazardous radioisotopes or expensive equipment) by their relatively low throughput, preventing an efficient screening by parallelization.

Thus, the field would benefit from a robust, fluorescent real-time assay that i) reports immediately the removal of the NAD-cap, ii) can be parallelized in a high-throughput manner using standard laboratory equipment, and iii) enables the discovery as well as characterization of NAD-RNA decapping enzymes.

**Figure 1:**
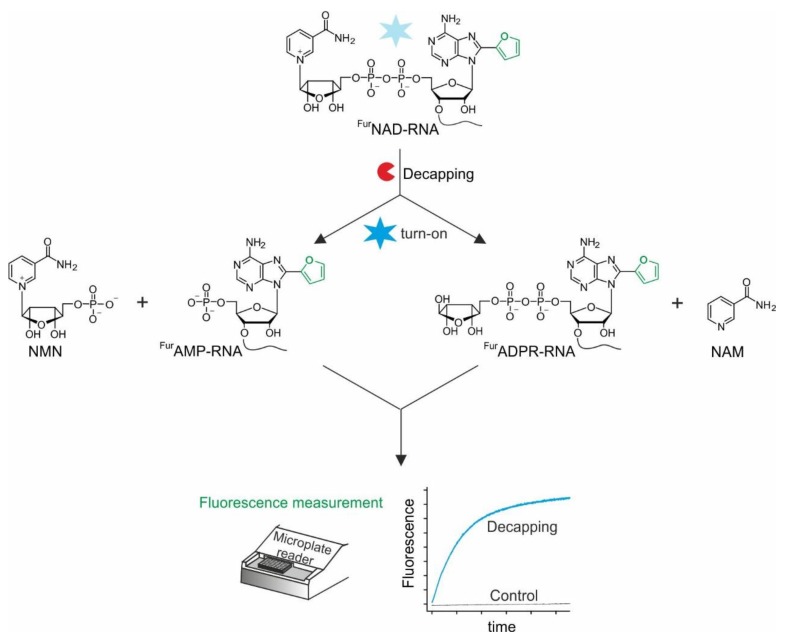
Fluorescence-based assay for NAD-RNA decapping using ^Fur^NAD-RNA. The decapping enzyme can remove NMN or NAM.

Here, we report the development of a highly sensitive, fluorescent system to identify novel NAD-RNA decapping enzymes based on a 8-(furan-2-yl)-NAD-RNA (^Fur^NAD-RNA). The design of the assay is shown in Figure 1. ^Fur^NAD-RNA, which is generated either by solid-phase oligonucleotide synthesis (SPOS) or in vitro transcription, is used as a substrate for putative decapping enzymes. In its intact form, the nicotinamide moiety efficiently quenches the fluorescence of the 8-(furan-2-yl)-adenosine. If an enzyme removes the quencher, a fluorescence increase can be detected in a standard cuvette photometer or a fluorescence microplate reader in real-time. In addition to characterizing several known NAD-RNA decapping enzymes, we report here the discovery of a novel decapping enzyme and chemistry, namely the removal of nicotinamide (NAM) by the human glycohydrolase CD38. 

## 2. Materials and Methods 

### 2.1. General Information

Reagents for oligonucleotide synthesis were purchased from Merck (Merck KGaA, Darmstadt, Germany) and used without further purification. Synthetic RNAs were purified by reversed-phase HPLC and their identities confirmed by MALDI-MS (oligonucleotides) or ESI-MS (initiator dinucleotides). DNA oligonucleotides (IVT templates) were purchased from Integrated DNA Technologies (IDT, Coralville, IA, USA).

### 2.2. Synthesis of 8-(furan-2-yl)-NAD 

The 8-substituted adenosine monophosphates were synthesized based on published protocols [28,29] with slight modifications: Final phosphoanhydride formation was accomplished via phosphate activation of nicotinamide mononucleotide (NMN) with 1,1’-carbonyldiimidazole (CDI) resulting in the corresponding 5’-phosphorimidazolide, Im-NMN [30]. Phosphorimidazolides are commonly used and show coupling efficiencies similar to morpholidates [31]. Coupling to the AMP derivatives (**2a**, **2b**) was performed in dry DMF at room temperature in the presence of excess MgCl_2_. Experimental details are highlighted in the supplementary information.

### 2.3. Synthesis of Oligonucleotides

Oligonucleotides were synthesized on an ExpediteTM 8909 automated synthesizer (Thermo Fisher Scientific, Waltham, MA, USA) using standard reagents from Merck (Proligo, Merck KGaA, Darmstadt, Germany) in a 1 µM scale. The synthesis of 8-(furan-2-yl)-adenosine-3’-phosphoramidite (**9**) is described in the supplementary information.

Short RNA strands were synthesized on solid-support (CPG), using standard conditions for RNA synthesis. The synthesis was carried out with commercial 5’-DMT-2′-O-TBDMS-(tac)-3’-phosphoramidites of all four canonical RNA nucleobases. For sequence information, see Table S1. For incorporation of the fluorescent nucleobase ^Fur^A at the 5’-end, the phosphoramidite (**9**) was coupled as the last nucleobase to the still immobilized, fully protected RNA strand. In the final step of the synthesis, commercial bis(2-cyanoethyl)-*N*,*N*-diisopropylphosphoramidite (Merck KGaA, Darmstadt, Germany) was used to generate the required 5’-phosphate. Oligonucleotides were deprotected and cleaved from the solid support with concentrated ammonia (1 mL, 28%) for 4 h at 40 °C, followed by three washes using 1 mL of water each time. The combined liquid phases were lyophilized. TBDMS-deprotection was carried out with a 600 µL mixture of 3HF*Et_3_N/DMF (1:1) and the oligonucleotides were precipitated with 1.2 mL isopropoxy-TMS and 5 mL Et_2_O and washed again with Et_2_O. Further reversed-phase HPLC purification resulted in the isolation of 5’-^Fur^AMP-RNAs (**RNA-1a** - **RNA-4a**, see Table S1), which were identified via MALDI-MS or ESI-MS (initiator-dinucleotides).

### 2.4. Synthesis of 5’-^Fur^NAD-RNAs

5’-NAD-RNAs (**RNA-1b** - **RNA-4b**, see Table S1) were prepared using previously reported phosphorimidazolide coupling with Im-NMN with the free phosphate of 5’-P-RNA in aqueous solution in the presence of MgCl_2_ (50–100 mM) at 50 °C [30]. 5’-P-RNA (10–100 nmol, 50–500 µM) prepared by solid-phase synthesis was incubated in the presence of a 100–500-fold excess of Im-NMN (50 mM, 1 mg per 50 µL reaction volume) and 50 Mm MgCl_2_ for 2 h at 50 °C. The concentration of Im-NMN should be kept constant, using 1 mg/50 µL reaction volume, as higher concentrations did not lead to a higher conversion. Pure 5’-^Fur^NAD-RNAs were obtained by reversed-phase HPLC purification. In most cases, two purification runs were necessary to obtain the required purity. The oligonucleotides were identified via MALDI-MS or ESI-HRMS for 5’-NAD-dinucleotides. 

### 2.5. HPLC Analysis and Purification

HPLC runs were conducted on an Agilent 1100 Series HPLC system (Agilent, Santa Clara, CA, USA) equipped with a diode array detector using a Phenomenex Luna 5 μm C18(2) 100 A (250 × 15 mm) (Phenomenex, Aschaffenburg, Germany) for oligonucleotides obtained from solid-phase synthesis or a Phenomenex Luna 5 μm C18(2) 100 A (250 × 4.6 mm) column for oligonucleotides generated by in vitro transcription using initiator nucleotides. The applied buffer system was 100 mM triethylammonium acetate pH 7.0 (buffer A) and 100 mM triethylammonium acetate in 80% acetonitrile (buffer B).

### 2.6. Mass Spectrometric Analysis

Small molecules were analysed with a Bruker microTOF-Q II ESI mass spectrometer. MS experiments for oligonucleotides were performed on a Bruker MicroFlex MALDI-TOF mass spectrometer (Bruker Corporation, Billerica, MA, USA) using 3-hydroxypicolinic acid (3-HPA) as matrix. For desalting oligonucleotides, ZipTip C18 pipette tips (Merck KGaA, Darmstadt, Germany) were used. 

### 2.7. In Vitro Transcription (IVT)

The sense and antisense ssDNA template strands (ssDNA, each 100 µM, from IDT; see Table S2) were mixed in a 1:1 ratio, incubated for 2 min at 90 °C before snap cooling on ice. Transcription was performed in a 50 µL scale in the presence of 5 µM DNA template (see Table S2), 2.5 mM initiator nucleotide (**DN-1a**–**DN-3a**) (the corresponding triphosphate was used in 1.5 mM concentration) 4 mM NTPs, 40 mM Tris-HCl pH 8.0, 1 mM spermidine, 22 mM MgCl_2_, 0.01% Triton X-100, 5% DMSO, 10 mM DTT, 0.25 µg/μL T7 RNAP and incubated for 4 h at 37 °C. RNA was purified by analytical reversed-phase HPLC.

### 2.8. Cloning of Different Nudix Hydrolases 

The genes encoding for *nudA*, *nudB*, *nudC* [3], *nudD*, *nudE*, *nudF, nudI, nudJ and nudL* were PCR-amplified from genomic DNA of *E. coli* K-12 (isolated via GenElute Bacterial Genomic DNA Kit, Merck KGaA, Darmstadt, Germany). The DNA sequence encoding for the *hNUDT5* gene was ordered from IDT and also amplified by PCR as well. pET28a-hDcp2 was ordered from Addgene (72214) (Addgene Europe, Teddington, UK). Restriction sites were introduced during amplification and resulting sequences are listed in Supplementary Table S3. The resulting PCR products were digested with XbaI/XhoI and NcoI, and cloned into pET-28c vector (Merck Millipore). After Sanger sequencing, the confirmed plasmids were transformed into *E. coli* One Shot BL21 (DE3) (Thermo Fisher Scientific, Waltham, MA, USA). 

### 2.9. Overexpression and Affinity Purification of Different Nudix Hydrolases 

*E. coli* One Shot BL21 (DE3) containing the respective plasmid were induced at OD_600_ = 0.8 with 1 mM IPTG. Bacteria were harvested after 3 h at 37 °C by centrifugation and lysed by sonication (30 s, 50% power, five times) in HisTrap buffer A (50 mM Tris-HCl pH 7.8, 1 M NaCl, 5 mM MgSO_4_, 5 mM 2-mercaptoethanol, 5% glycerol, 5 mM imidazole, 1 tablet per 500 mL cOmplete EDTA-free protease inhibitor cocktail (Merck KGaA, Darmstadt, Germany). For the preparation of NudC, 1 M urea was added to HisTrap buffer A and B to remove contaminating RNA [32]. The lysate was clarified by centrifugation (37,500× *g*, 30 min, 4 °C) and the supernatant was applied to a Ni-NTA HisTrap column (GE Healthcare Europe GmbH, Freiburg, Germany). The protein was eluted with a gradient of HisTrap buffer B (HisTrap buffer A with 300 mM imidazole) and analysed by SDS–PAGE. Fractions of interest were concentrated in Amicon filters (Merck KGaA, Darmstadt, Germany, CO 10 kDa, centrifugation at 5000× *g*, 4 °C). The enzymes were additionally purified via size exclusion chromatography with a HiPrep 16/60 Sephacryl S-200 High Resolution column (GE Healthcare Europe GmbH, Freiburg, Germany) (buffer: 50 mM Tris-HCl pH 8.0, 300 mM NaCl) and stored at −20 °C in 25 mM Tris-HCl pH 8.0, 150 mM NaCl, 50% glycerol. All purified protein samples were 95% pure, according to SDS–PAGE.

### 2.10. Fluorescence Spectroscopy Using a Sensitive Cuvette Spectrofluorometer

Fluorescence time course and fluorescence spectra were measured with a FP-6500 spectrofluorometer (JASCO Deutschland GmbH, Pfungstadt, Germany) at 37 °C. Reactions were performed in a 20 µL scale in the presence of ^Fur^NAD (4.5 µM) or ^Fur^NAD-RNA (4.5 µM), 25 mM Tris-HCl pH 7.5, 50 mM NaCl, 50 mM KCl, 10 mM MgCl_2_, 1 mM DTT.

The following settings were applied for fluorescence measurements: sensitivity mode: manual 350 V; λ_exc/em_: 304/380 nm; 5 nm excitation/emission bandwidth, response: 0.5 s; at 37 °C.

All enzymes were applied in low µM concentrations (4 µM) except for CD38 (2.5 nM), which was highly active on modified NAD as well as NAD-RNAs.

### 2.11. Fluorescence Time Course Measurements Using a Fluorescence Microplate Reader 

Reactions were performed in a 20 µL scale using a 384 well-plate format (UV-permeable from Greiner, UV-Star) in the presence of ^Fur^NAD (4.5 µM) or ^Fur^NAD-RNA (4.5 µM), 25 mM Tris-HCl pH 7.5, 50 mM NaCl, 50 mM KCl, 10 mM MgCl_2_, 1 mM DTT and an appropriate enzyme. Enzymes were either cloned, expressed and purified as described and their concentration determined using Nanodrop, or obtained from commercial sources (CD38 (Sino Biological, Inc., Wayne, USA), RppH (New England Biolabs GmbH, Frankfurt am Main, Germany), polyphosphatase (Biozym Scientific GmbH, Hessisch Oldendorf, Germany), ADP ribosyl cyclase (ADPRC) (Merck KGaA, Darmstadt, Germany)).

Time course measurements of fluorescence intensity were conducted using a Tecan Sapphire 2 fluorescence microplate reader (Tecan Group Ltd., Männedorf, Switzerland. The following settings were applied for fluorescence measurements: high sensitivity mode; λ_exc/em_: 304/380 nm; 20 nm excitation/emission bandwidth; measurement position: bottom of the well plate; gain 75; 40 µs integration time; measurement every 20 s; at 37 °C. 

### 2.12. Radioactive Validation of CD38 Activity 

5’-radioactively labelled NAD-RNAI was prepared by IVT as described in [24]. Briefly, 5’-PPP-RNAs, prepared by standard IVT were converted into 5’-monophosphate RNA using RNA 5’-polyphosphatase (Biozym Scientific GmbH, Hessisch Oldendorf, Germany). 5’-P-RNA was purified by phenol-chloroform/diethyl ether extraction. After isopropanol precipitation, 5’-P-RNA was converted into 5’-^32^P-RNA using T4 polynucleotide kinase (PNK) in 1 x reaction buffer B and 250 μCi ^32^P-γ-ATP (Hartmann Analytic, Braunschweig, Germany). The reaction was incubated at 37 °C for two hours and purified by phenol-chloroform/diethyl ether extraction and isopropanol precipitation. To convert the purified 5’-^32^P-RNAs into 5’-^32^P-NAD-capped RNAs, 5’-^32^P-RNAI was incubated with 1000-fold excess of Im-NMN in the presence of 50 mM MgCl_2_ and at 50 °C for 2 hours [30]. 5’-^32^P-NAD-capped RNAs were purified from remaining 5’-^32^P-RNAs by acryloylaminophenyl boronic acid (APB) gel electrophoresis [24] and isopropanol precipitated.

Enzyme kinetics of CD38 were performed in the presence of 25 mM Tris-HCl pH 7.5, 50 mM NaCl, 50 mM KCl, 10 mM MgCl_2_, 1 mM DTT, 50 nM 5’-^32^P-NAD-RNAI and 3 nM CD38. Enzymatic reactions were stopped by heating the samples to 98 °C for 30 s and RNA digested to single nucleotides by Nuclease P1 (Merck KGaA, Darmstadt, Germany) treatment (0.1 U/µL). CD38-mediated conversion of ^32^P-labelled NAD-RNA was analysed by TLC using 4:6 1 M ammonium acetate (pH 5.5)/ethanol as mobile phase as previously described [26]. 1 μL of each sample was applied to TLC Alugram XtraSil G/UV254 (Macherey-Nagel, Düren, Germany) plates and run until the mobile phase reached a line 1 cm below the upper end of the TLC plate.

## 3. Results

### 3.1. Chemo-Enzymatic Synthesis of ^Fur^NAD and ^Fur^NAD-RNA

For a fluorescent turn-on NAD-RNA decapping assay, we first had to develop fluorogenic NAD-RNA derivatives that are accepted as substrates by putative decapping enzymes. We were inspired by earlier work from the Wagner group who developed fluorogenic substrates for studying NAD hydrolysis and related reactions [28]. These authors found that the addition of 5-membered heterocycles, in particular pyrrole, to the 8-position of the adenine of NAD resulted in fluorogenic derivatives, in which the 8-(pyrrol-2-yl)-adenosine was the fluorophore, whereas the nicotinamide moiety acted as an efficient fluorescence quencher, likely by contact quenching. These pyrrolyl-NAD derivatives were readily accepted by three different enzymes, namely a pyrophosphohydrolase, a glycohydrolase and an ADP-ribosyl cyclase (ADPRC), which removed the quencher (either in the form of NAM or as NMN) from the vicinity of the fluorophore and thereby caused a fluorescence increase [28]. 

**Figure 2:**
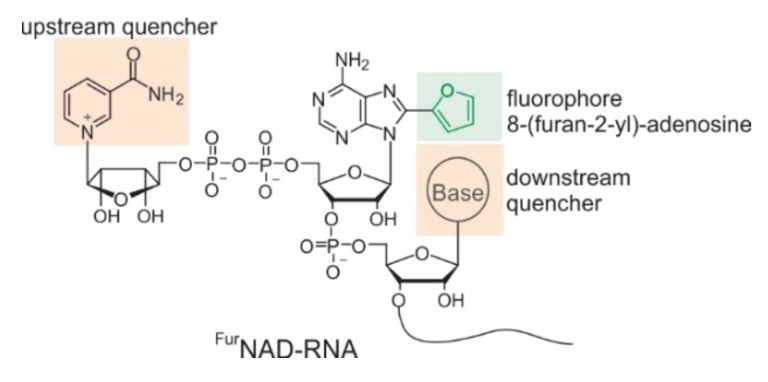
Illustration of ^Fur^NAD-RNA.

Based on these findings, we considered it reasonable to assume that the installation of a similar fluorescent substitution at the 5’-end of NAD-RNA should also lead to turn-on probes that might report the action of decapping enzymes. In contrast to the previously mentioned design in which the fluorophore is inactivated by one quencher, we expected that the fluorophore will interact with two quenching moieties in NAD-RNA, namely the nicotinamide “upstream” and the following nucleobase “downstream” (Figure 2).

To identify the most suitable moiety to be linked to adenosine, we attached five different heterocycles to the 8-position of AMP, namely 2-furan, (^Fur^AMP, **2a**) 2-pyrrole (^Pyr^AMP, **2b**), 2-thiophene (^Thio^AMP, **2c**), 2-(5-formylfuran, ^ForFur^AMP, **2d**), and 2-(5-formylthiophene, ^ForThio^AMP, **2e**) (Figures S1 and S2) and recorded their fluorescence emission spectra (Figure S3). Only compounds **2a**, **2b** and **2c** showed significant fluorescence, and the addition of a formyl group had a detrimental effect on the fluorescence. 

**Figure 3:**
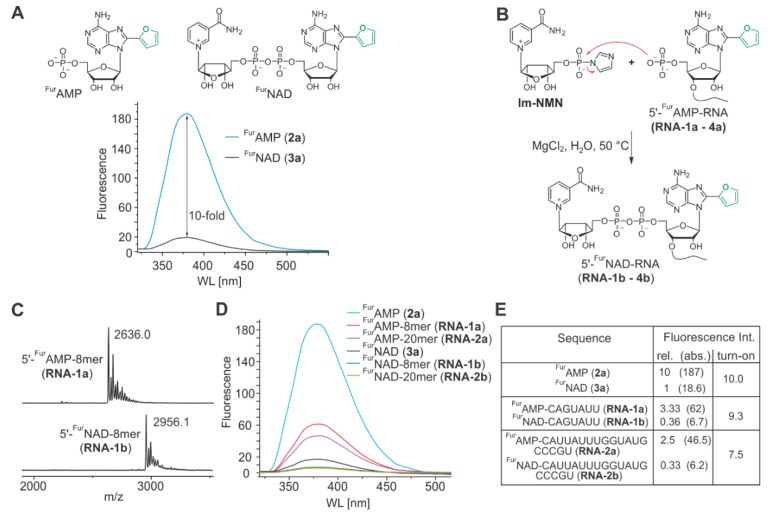
Synthesis and characterization of 8-(furan-2-yl)-NAD-RNAs. (**A**) Fluorescence properties of 8-(furan-2-yl)-AMP (**2a**) and 8-(furan-2-yl)-NAD (**3a**), with 10-fold higher fluorescence intensity for **2a**. (**B**) Synthesis of 5’-^Fur^NAD-RNA (**RNA-1b–RNA-4b**, sequences in Table S1) via coupling of 5’-P-RNA (**RNA-1a–RNA-4a**) with Im-NMN. (**C**) MALDI-MS of **RNA-1a** and **RNA-1b**; m/z calculated for (M + H)^+^, 2640.4 (RNA-1a) and (M)^+^, 2956.4 (**RNA-1b**). (**D**,**E**) Sequence and fluorescence properties of 8-(furan-2-yl)-AMP-RNAs and corresponding 8-(furan-2-yl)-NAD-RNAs.

^Fur^AMP (**2a**, [33]) had the highest fluorescence emission. Compounds **2a**, **2b** and **2c** had very similar absorption maxima (300–308 nm), while the emission maxima varied between 374 and 405 nm. We therefore synthesized 8-(furan-2-yl)-NAD (^Fur^NAD, **3a**) and the previously described 8-(pyrrol-2-yl)-NAD (^Pyr^NAD, **3b**) (Figure S1). After the coupling of AMP derivatives ^Fur^AMP and ^Pyr^AMP with Im-NMN to furnish the NAD structure [30], ^Fur^NAD and ^Pyr^NAD were obtained in yields of 31% and 28%, respectively. Compared to the corresponding ^Fur^AMP and ^Pyr^AMP derivatives, both NAD derivatives showed a ~10-fold fluorescence quenching effect (Figure 3A, Figure S4). The location of the absorption and emission maxima remained unchanged by linkage to NMN (^Fur^NAD 304/380 nm; ^Pyr^NAD 308/374 nm). 

After identifying ^Fur^NAD and ^Pyr^NAD as promising candidates at the nucleotide level, we had to develop methods to synthesize these as 5’-cap structures of longer RNA strands. The direct incorporation of **3a** by T7 RNA polymerase during in vitro transcription was considered [30,34], but in contrast to NAD, the enzyme did not accept this substrate, likely due to the rigid and bulky nature of the heterocyclic substituent (data not shown) [35]. On the other hand, the direct formation of pyrophosphate bonds during solid-phase oligonucleotide synthesis (SPOS) is also far from trivial. We therefore applied our previously reported two-step process combining SPOS with phosphoimidazolide coupling in solution. Therefore, we first synthesized the 3’-phosphoramidite of 8-(furan-2-yl)-adenosine (**9**) (Figure S5). During automated synthesis, this phosphoramidite was attached as the last nucleotide, followed by 5’-phosphorylation. This resulted in RNAs with ^Fur^AMP at the 5’-terminus. Four 5’-^Fur^AMP-RNAs of different length and nucleotide sequence (**RNA-1a–RNA-4a**) were synthesized, deprotected, and purified by HPLC (Figure S6A, Table S1). Finally, the nicotinamide moiety was attached by coupling nicotinamide riboside 5’-phosphoimidazolide (Im-NMN) as described previously for conventional NAD-RNA [30] (Figure 3B). ^Fur^NAD-RNAs were finally purified by HPLC (Figure S6B).

Purified oligonucleotides (**RNA-1b–RNA-4b**) bearing a 5’-^Fur^NAD modification were obtained in moderate yields (30–60%) and their identities confirmed by MALDI-MS (Figure 3C and Figure S6C) and affinity gel electrophoresis (Figure S6D) [24]. Thus, SPOS combined with phosphoimidazolide coupling allowed the efficient and reliable synthesis of short- and medium-length ^Fur^NAD-RNA strands. 

Solid-phase synthesis is, however, not efficient for the preparation of strands > 50 nt, and many NAD-RNAs found in nature to date are > 50 nt. To allow the synthesis of 5’-^Fur^NAD-RNAs of variable lengths, in vitro transcription (IVT) was revisited. As neither ^Fur^NAD (see above) nor ^Fur^AMP were accepted as a substrates by T7 RNA polymerase (data not shown), we modified the approach using initiator dinucleotides, which we had previously employed to solve the problem of “unincorporatable” 5’-moieties [36]. Thus, three dinucleotides (DN) ^Fur^AA (**DN-1a**), ^Fur^AG (**DN-2a**) and ^Fur^AU (**DN-3a**), were synthesized by SPOS (Figure S7) and subjected to IVT using T7 RNA polymerase to generate ^Fur^AMP-RNAs (Figure S8, 25mer A/G/U-DNA templates see Supplementary Table S2). ^Fur^AA and ^Fur^AG were incorporated with efficiencies of 45% and 37% respectively (Figure S8B). In vitro transcribed ^Fur^AMP-RNAs were purified by HPLC. MALDI-MS of full-length RNA transcripts and respective controls (5’-pppRNA) confirmed the synthesis of pure ^Fur^AMP-RNAs (Figure S8C,D). 

### 3.2. Fluorescence Properties of ^Fur^NAD-RNAs

The main difference between ^Fur^NAD and ^Fur^NAD-RNA is that the fluorophore 8-(furan-2-yl)-adenine is quenched by one moiety (nicotinamide) from one side in the first case, and potentially by two (nicotinamide plus the neighbouring nucleobase) from both sides in the latter (Figure 2). Thus, a lower overall fluorescence of ^Fur^NAD-RNA, compared to ^Fur^NAD, is expected. To analyse the effect of the downstream base, we prepared ^Fur^NAD-RNAs as well as ^Fur^AMP-RNA carrying an abasic site (referred as ^Fur^X-Rsp-X, Figure S8E) and determined their fluorescence properties. In the presence of an abasic site (**RNA-3a**, **RNA-3b**), fluorescence intensities were in the same range as determined for the non-modified ^Fur^AMP-RNAs or ^Fur^NAD-RNAs (**RNA-1a**, **RNA1-b**). 

Furthermore, the identity of the neighbouring nucleobase is expected to influence fluorescence quenching. Indeed, fluorescence spectroscopy on ^Fur^AMP-dinucleotides ^Fur^AA (**DN-1a**), ^Fur^AG (**DN-2a**), ^Fur^AU (**DN-3a**), and ^Fur^AC (**DN-4a**) revealed the influence of the neighbouring base: Quenching was strongest for G (23% remaining fluorescence, relative to the ^Fur^AMP mononucleotide), followed by C and U (33%), while A showed the weakest quenching (69%) (Figure S9). Attachment of the nicotinamide moiety to ^Fur^AMP-RNAs to generate ^Fur^NAD-RNAs caused 7.5–10-fold quenching, as observed for the nucleotides (Figure 3D,E). 

**Figure 4:**
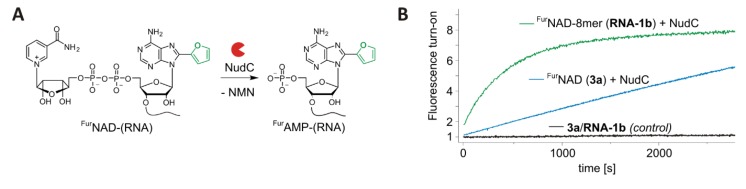
Application of ^Fur^NAD-(RNA) as fluorescent turn-on probes. (**A**) Enzymatic decapping of ^Fur^NAD-(RNA) by NudC. (**B**) Enzyme kinetics of NudC in the absence (control) and the presence of ^Fur^NAD (4.5 µM) or ^Fur^NAD-RNA (4.5 µM). NudC shows highest activity on RNA as substrate.

Thus, ^Fur^NAD-RNA appears to be suitable for turn-on decapping assays. To demonstrate this property, we analysed the well-described NAD-RNA decapping activity of the bacterial Nudix hydrolase NudC [32,37] using an 8mer ^Fur^NAD-RNA as a substrate. This enzyme hydrolyses the pyrophosphate bond within the NAD (or here ^Fur^NAD) moiety, thereby releasing the quencher NMN and ^Fur^AMP-RNA. Indeed, the fluorescence time traces (Figure 4 and  S10) show an increase of the fluorescence signal over time, while in the no-enzyme controls the signal remains constant (Figure 4B). Comparison of same amounts of 8mer ^Fur^NAD-RNA (Figure 4B, green trace) with ^Fur^NAD (blue trace) is in agreement with our previous findings that NudC prefers NAD-RNA over NAD [3,32].

### 3.3. Screening for Novel NAD-RNA Decapping Enzymes

**Figure 5:**
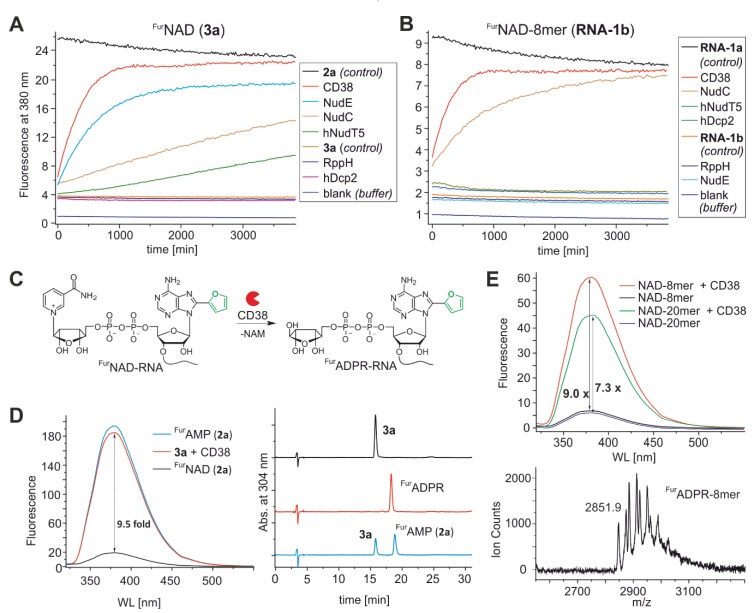
Identification and characterisation of novel decapping enzymes using ^Fur^NAD and ^Fur^NAD-RNAs. Different enzymes were tested for their activity on (**A**) ^Fur^NAD (**3a**, 4.5 µM) and (**B**) ^Fur^NAD-8mer (**RNA-1b**, 4.5 µM). Fluorescence time course measurements were performed using a microplate reader (λ exc 304 nm/em 380 nm). (**C**) Schematic representation of the conversion of ^Fur^NAD-RNAs into ^Fur^ADPR-RNAs by CD38. (**D**) Comparison of fluorescence intensities and HPLC analysis of the reaction of CD38 (2.5 nM) with ^Fur^NAD after 45 min reaction; retention times of 3a (15.8 min), ^Fur^ADPR (18.2 min), 2a (18.8 min) (Phenomenex Luna 5u, 25 × 4.6 mm, 1 mL/min, 5–15% buffer B in 30 min). (**E**) Immediate fluorescence turn-on after addition of CD38 (50 nM) to 5’-^Fur^NAD-RNAs (each 4.5 µM; 8mer-, **RNA-1b**, 20mer-, **RNA-2b**) and corresponding MALDI-MS of ^Fur^ADPR-8mer: calculated *m/z:* 2852.36. Corresponding HPLC chromatograms are shown in Figure S13.

Having established the feasibility of the ^Fur^NAD-RNA system with a known NAD-RNA decapping enzyme, we now investigated its capacity to discover novel decapping enzymes. To this end, we applied a library of different Nudix hydrolases, deglycosylases, and other enzymes to ^Fur^NAD and ^Fur^NAD-RNA, and monitored the increase of the fluorescent signal in real time on a fluorescence microplate reader. Using ^Fur^NAD, the assay revealed a fluorescence increase for all enzymes known to hydrolyse NAD into AMP or ADP-ribose, namely the human cluster of differentiation 38 (CD38) glycohydrolase [38], *E. coli* NudE [39], and with much lower activity NudC, ADP ribosyl cyclase (ADPRC) from *Aplysia californica* [40], and human hNUDT5 [41] (Figure 5A and Figure S11A). 

We screened the same enzyme library on ^Fur^NAD-RNA (Figure 5B and Figure S11B). In addition to the positive control NudC, we observed a marginal fluorescence increase for ADPRC (which was expected, as we used this enzyme previously for tagging NAD-RNAs for selective capture and sequencing [3,5]). Unexpectedly, the strongest fluorescence was recorded for human CD38, (at least twice as fast as NudC under comparable conditions). CD38, a type II membrane protein, was originally described to have a widespread distribution in tissues and to synthesize both cyclic ADP ribose (cADPR) and nicotinic acid adenine dinucleotide phosphate (NAADP) [38,42]. It was originally reported to be an ectoenzyme (which was later questioned [43,44]), but a comprehensive picture of its in vivo roles is only beginning to emerge. Importantly, this enzyme has never been described in any context related to RNA. Our novel assay reveals that in vitro, CD38 efficiently processes ^Fur^NAD-RNA, likely by converting it into ^Fur^ADPR-RNA and nicotinamide (Figure 5). The formation of this novel 5’-RNA-modification, ADPR-RNA, was verified by MALDI-MS (Figure 5E and Figure S12).

In addition, this assay confirmed the strikingly different specificities of *E. coli* Nudix hydrolases NudC and NudE [32]: While NudC prefers NAD-RNA over NAD, NudE is much more efficient in hydrolysing NAD than NudC but does not accept NAD-RNA as substrate. ADPRC appears to process ^Fur^NAD and ^Fur^NAD-RNA at similar rates. The human enzyme hNUDT5 hydrolyses only ^Fur^NAD, but not ^Fur^NAD-RNA despite its reported broad substrate specificity [45]. On the other hand, typical RNA-processing enzymes such as RppH, RNA polyphosphatase, or human Dcp2 were found to be inactive on ^Fur^NAD-RNA, in agreement with independent observations on NAD-RNA [10] and in agreement with the assumption of NAD capping being an orthogonal strategy for stabilizing RNA against degradation [3,46]. 

### 3.4. Determination of Kinetic Parameters

To explore the sensitivity of our turn-on ^Fur^NAD-RNA assay and to increase its versatility, we applied it to the determination of enzyme-kinetic parameters (K_m_ and k_cat_). CD38 was originally described to modulate NAD concentration by hydrolysing NAD into ADPR or cADPR at very low µM concentrations, using a fluorescence-based enzymatic assay using 1,N^6^-ethenonicotinamide adenine dinucleotide (ε-NAD) and nicotinamide guanine dinucleotide (NGD) as substrates, respectively [47,48].

First, ^Fur^NAD was added to CD38, and the initial conversion rates were determined at varying substrate concentrations, yielding a curve that showed Michaelis-Menten saturation. The decline of the initial rates at high substrate concentration was suggestive of substrate inhibition (Figure S14A,B), and fitting the data point to the appropriate equation yielded with a K_m_ value of 9.34 µM, an inhibition constant K_i_ of 84 µM, and a k_cat_ of 5.8 s^−1^ (Figure S14C). These values are consistent with previously published K_m_ values, ranging for the natural substrate NAD from 14 to 48 µM [47,49]. 

For the ^Fur^NAD-RNA 8mer, we measured a K_m_ of 23.4 µM, Ki of 100 µM, and a k_cat_ of 21 s^−1^. Thus, despite a slight drop in binding affinity relative to ^Fur^NAD, the ^Fur^NAD-RNA 8mer is converted nearly three times faster. 

To ensure that the observed fluorescence increase upon CD38 addition is not an artefact induced by the presence of the unnatural furanyl substituent, we used a conventional, TLC-based assay [32] with conventional NAD-RNA. This NAD-RNA carried a site-specific ^32^P label within the NAD moiety. After processing by CD38, reaction mixtures were digested to nucleotides by nuclease P1, liberating radioactive ADPR (from converted product RNA) or NAD (from unconverted substrate RNA). Separation by TLC and densitometric analysis by phosphorimaging allowed quantification (Figure S15). This analysis confirmed the rapid decapping of NAD-RNA in a time frame comparable to the ^Fur^NAD-RNA (compare to Figure 5). Moreover, we determined the processing of NAD-RNA into ADPR-RNA by CD38 in the presence of a 20- and 200-fold excess of NAD over NAD-RNA. This experiment shows that a 200-fold excess of NAD over NAD-RNA is needed to efficiently suppress the formation of ADPR-RNA (Figure S15B–E). At a 20-fold excess of NAD, the reaction kinetics are only slowed down by a factor of two. These findings further support the observation that CD38 - at least in vitro - favours NAD-RNA as a substrate over NAD.

## 4. Discussion

The discovery of NAD-capped RNAs in bacteria [3,8,9], mammalian cells, yeast [11,18] and plants [12,13] highlights the importance of studying the biogenesis, function, and removal of novel 5’-RNA modifications. The identification of new decapping enzymes can make important contributions to understanding the biological roles of NAD-capped RNAs [50].

In this work, we present a sensitive fluorescence turn-on system to discover and to characterize NAD-RNA decapping enzymes based on ^Fur^NAD-RNA. ^Fur^NAD-RNA, prepared either by SPOS or by IVT, is used as a substrate for putative decapping enzymes. Removal of the quencher by the latter, NAM or NMN, leads to a real-time increase in fluorescence, and this assay can be easily parallelized by using a fluorescence microplate reader.

**Figure 6:**
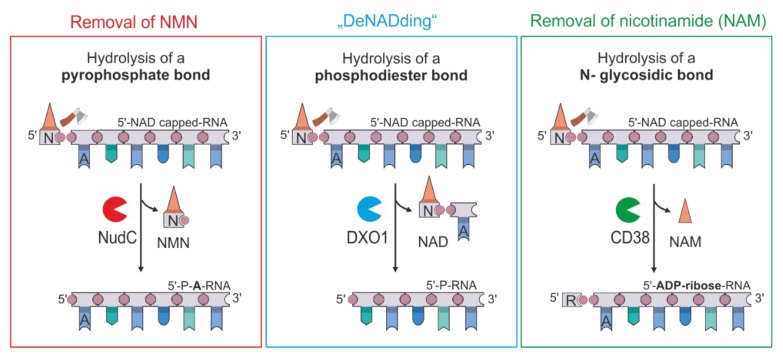
Decapping of NAD-RNAs by different mechanisms. Mechanism I: Hydrolysis of the pyrophosphate bond of NAD by Nudix hydrolases such as NudC or BsRppH. NAD-RNA is processed to 5’-monophosphorylated RNA, which contains the adenosine of NAD as the first nucleotide (5’-P-A-RNA) and NMN. Mechanism II: “DeNADding”—Removal of the NAD-cap by DXO1. The phosphodiester bond between NAD and the second nucleotide is hydrolysed, which releases NAD and shortened 5’-monophosporylated RNA. Mechanism III: Removal of nicotinamide by hydrolysis of the N-glyosidic bond: CD38 catalyses the removal of nicotinamide (NAM), thereby converting NAD-RNA into ADPR-RNA.

The ^Fur^NAD-RNA assay led to the discovery and characterization of a hitherto unknown putative decapping enzyme, CD38, which efficiently decaps NAD-RNA in vitro. This human enzyme uses a decapping chemistry entirely different from the two known classes of NAD decapping enzymes, as it converts NAD- RNA into 5’-ADP-ribose-RNA, liberating nicotinamide (Figure 6). In contrast, the prokaryotic decapping enzymes NudC and BsRppH [3,8,32] and the putative eukaryotic NAD-decapping enzyme Npy1 [51] hydrolyse the pyrophosphate bond within NAD, while DXO enzymes take off the entire NAD cap [10,18,21]. Interestingly, Npy1p, DXO1 and CD38 are differently localized in the cell. While the NudC homolog Npy1 has been reported as a peroxisomal membrane protein in yeast [52], DXO1 is mainly localized in the cytoplasm [53], and the related Rai1 is a nuclear protein [54]. CD38, however, is a membrane protein that is embedded in the plasma membrane of certain mammalian cells, in particular immune cells. Originally described as an ectoenzyme (with the catalytic site located outside the cell), it has later been proposed to exist in two opposing orientations, which are based on “flipping” its catalytic domain from the outside to the inside of the cell, thereby regulating its signalling activity [44,55]. However, further research is necessary to confirm the NAD-RNA decapping activity of CD38 in vivo. The observations that a) at least four different (putative) NAD-RNA decapping enzymes exist (Figure 6); b) they use at least three different mechanisms; and c) they are localized in different cell types and/or cellular compartments suggest that NAD-RNA decapping is an essential and regulated activity in eukaryotes.

## 5. Conclusions

The application of synthetic light-up NAD-RNA presented in this article will facilitate and promote the elucidation of the biological relevance of NAD capping in prokaryotes and eukaryotes, the identification of binding partners, players and mechanisms in decapping and RNA turnover.

Using ^Fur^NAD-RNAs, we discovered that the eukaryotic glycohydrolase CD38 processes NAD-capped-RNA in vitro into ADP-ribose-modified-RNA and nicotinamide and therefore might act as a decapping enzyme in vivo. The existence of multiple decapping pathways suggests that the removal of the NAD-cap is an important and regulated process in eukaryotes.

## Supplementary Materials

The following are available online at https://www.mdpi.com/2218-273X/10/4/513/s1, Figure S1: Synthesis of 8-substituted fluorescent AMP (2a–2e) and NAD analogs (3a,3b). Figure S2: HRMS analysis of 8-substituted AMP (2a,2b) and NAD (3a,3b) derivatives. Figure S3: Emission spectra of synthesised 8-Ar-AMP derivatives after irradiation at absorption maxima. Figure S4: Comparison of fluorescence intensities of NAD analogs 3a (exc. 304/em. 380 nm) and 3b (exc. 308/em. 374 nm) and corresponding AMP analogs 2a and 2b. Figure S5: Synthesis of 8-(furan-2-yl)-adenosine (9) for incorporation into RNA. Figure S6: Purification and validation of synthesized 20mer RNAs. Figure S7: HRMS (ESI-TOF, negative), HPLC analysis and identification of synthesised dinucleotides (DN-1a–DN-4a and DN-1b–DN-4b). Figure S8: Enzymatic ^Fur^AMP-RNA synthesis purification and analysis. Synthesis of 5’-8-(furan-2-yl)-AMP 26/27mer RNAs via IVT using initiator dinucleotide. Figure S9: Impact of the neighbouring base upon ^Fur^AMP fluorescence. Figure S10: Fluorescence time-course measurement using 8-(furan-2-yl)-NAD (3a, 4.5 µM) and 8-(furan-2-yl)-NAD-RNA (RNA-1b, 4.5 µM) in the presence of NudC (4 µM). Figure S11: Screening of enzymes that hydrolyse FurNAD or remove the NAD-cap. Figure S12: Identification of CD38 reaction products by MALDI-MS. Figure S13: HPLC analysis of the hydrolysis of NAD-8mer and ^Fur^NAD-8mer by CD38. Figure S14: Determination of the kinetic parameters of CD38 (2.5 nM) in the presence of different concentrations of ^Fur^NAD and ^Fur^NAD-RNAs. Figure S15: Validation of CD38 activity and specificity using radioactive 5’-NAD-capped RNA. Figure S16: ^1^H-NMR spectrum of 3a. Figure S17: ^13^C-NMR spectrum of 3a. Figure S18: ^31^P-NMR spectrum of 3a. Figure S19: ^1^H-NMR spectrum of the phosphoramidite 9. Figure S20: 13C-NMR spectrum of the phosphoramidite 9. Figure S21: 31P-NMR spectrum of the phosphoramidite 9. Supplementary Table S1. Sequences of RNAs synthesized by solid-phase oligonucleotide synthesis. Supplementary Table S2. Synthesized dinucleotides, which were incorporated into the 5’-end of different 25mer RNAs using indicated ds 25mer DNA templates. Supplementary Table S3: DNA sequence of cloned enzymes, used in this study.
