# Supplementary material for: A Novel NAD-RNA Decapping Pathway Discovered by Synthetic Light-Up NAD-RNAs
Source: Biomolecules. 2020 Mar 28;10(4):513. doi: 10.3390/biom10040513 (PMC7226252; doi:10.3390/biom10040513)
Supplement: Supplementary file 1 [file biomolecules-10-00513-s001.pdf]

Supplementary Information

A novel NAD-RNA decapping pathway discovered by synthetic light-up NAD-RNAs

Florian Abele [1, †], Katharina Höfer [1, †], Patrick Bernhard [1], Julia Grawenhoff [1], Maximilian Seidel [1], André Krause [1], Sara Kopf [1], Martin Schröter [1] and Andres Jäschke [1,*]

[1] Institute of Pharmacy and Molecular Biotechnology, Heidelberg University, Im Neuenheimer Feld 364, Heidelberg, 69120, Germany

* Correspondence: jaeschke@uni-hd.de; Tel.: (+49-6221-544851)

† These authors contributed equally to this work.

Table of Contents

Figures S1-S21	Page S2-S22
Chemical synthesis	Page S23-S35
Tables S1-S3	Page S36-S40

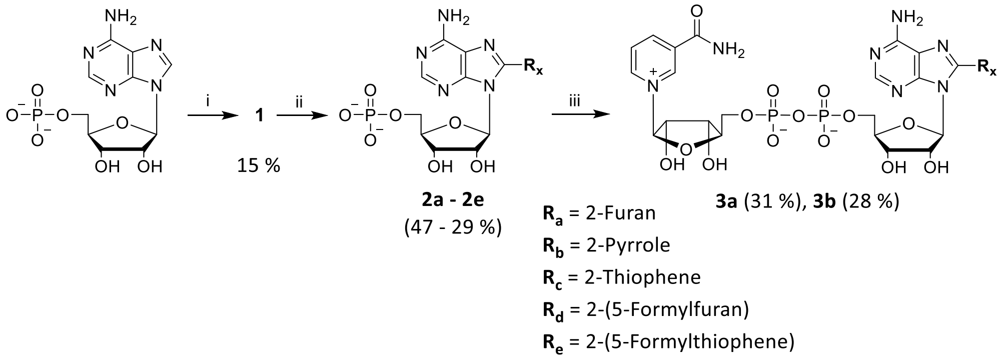

Figure S1: Synthesis of 8-substituted fluorescent AMP (**2a – 2e**) and NAD analogues (**3a, 3b**). Reagents and conditions: i) Br_2, aq. KH_2PO_4 (pH 5), rt, 16 h; ii) R-$B(OH)_2$ (1.5 eq), $Pd(OAc)_2$ (3 mol %), TPPTS (7 mol %), K_2CO_3 (2 eq), H_2O, 80 °C, 2 h; iii) Im-NMN, $MgCl_2$ (10 eq) DMF, rt, 5 h.

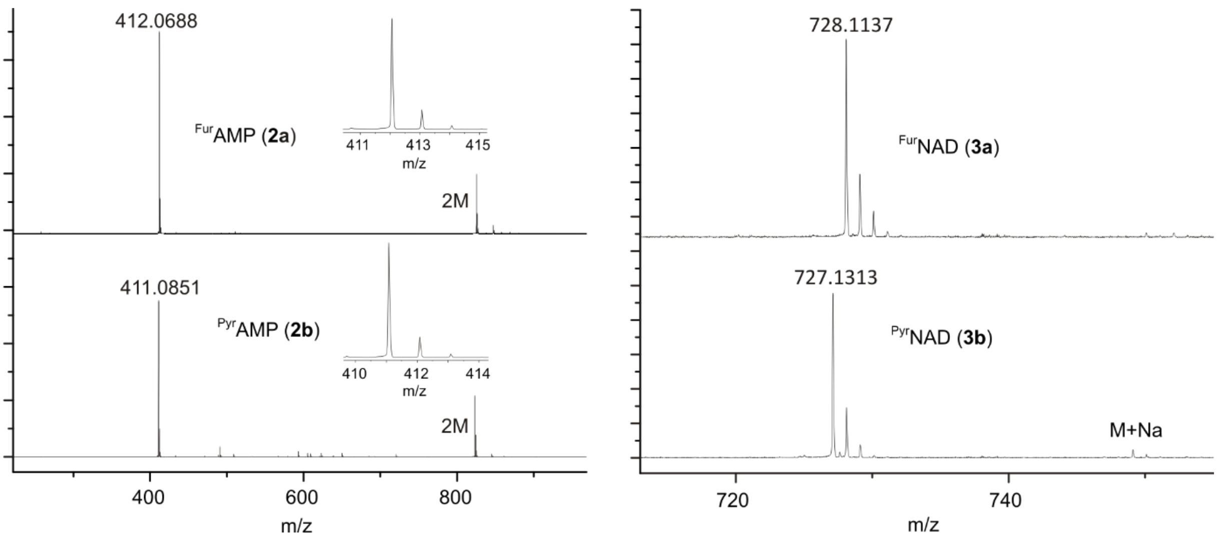

Figure S2: HRMS analysis of 8-substituted AMP (**2a, 2b**) and NAD (**3a, 3b**) derivatives.

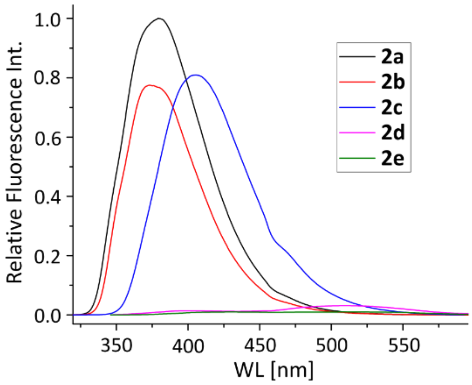

	2a	2b	2c	2d	2e
λmax Absorption [nm]	304	308	300	338	337
λmax Emission [nm]	380	374	405	401/ 509	432/ 513

Figure S3: Emission spectra of synthesised 8-Ar-AMP derivatives after irradiation at absorption maxima. Substituents at the adenine 8-position: a) 2-furan; b) 2-pyrrole; c) 2-thiophene; d) 2-(5-formylfuran); e) 2-(5-formylthiophene).

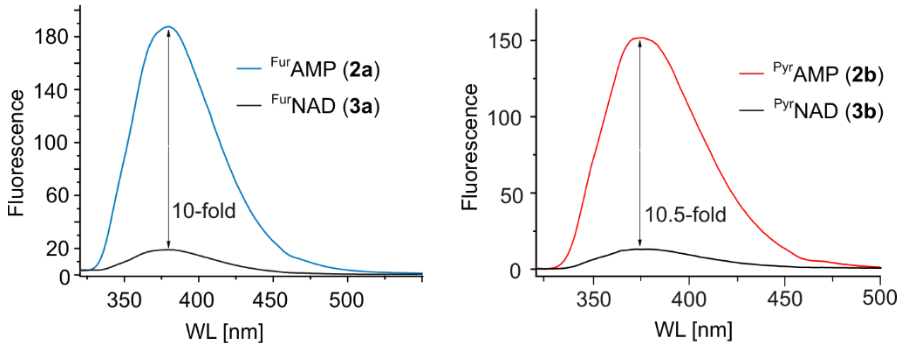

Figure S4: Comparison of fluorescence intensities of NAD analogues **3a** (exc. 304/em. 380 nm) and **3b** (exc. 308/em. 374 nm) and corresponding AMP analogues **2a** and **2b**.

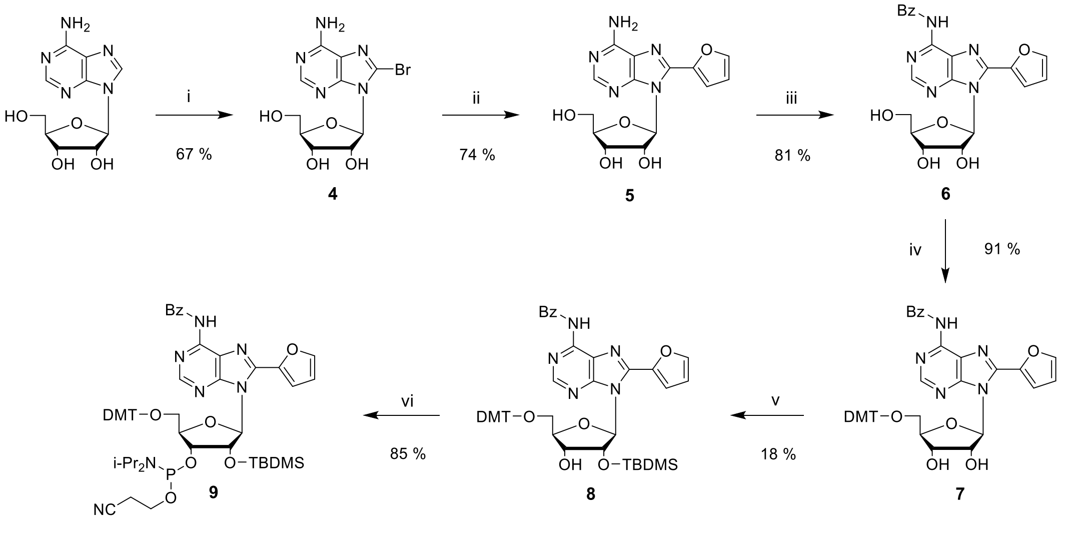

Figure S5: Synthesis of 8-(furan-2-yl)-adenosine phosphoramidite (**9**) for incorporation into RNA. Reagents and conditions: i) Br_2, RT, 24 h AcOH/NaOAc (pH 4); ii) Na_2PdCl_2, TPPTS, K_2CO_3, H_2O, 80°C, 2 h; iii) TMSCl, Bz-Cl, pyridine, RT, 2 h; iv) DMTCl, DMAP, pyridine, 4 h, RT; v) TBDMSCl, $AgNO_3$, THF, pyridine, RT, 4 h; vi) CEPCl, Hünig's Base, THF, RT, 2 h.

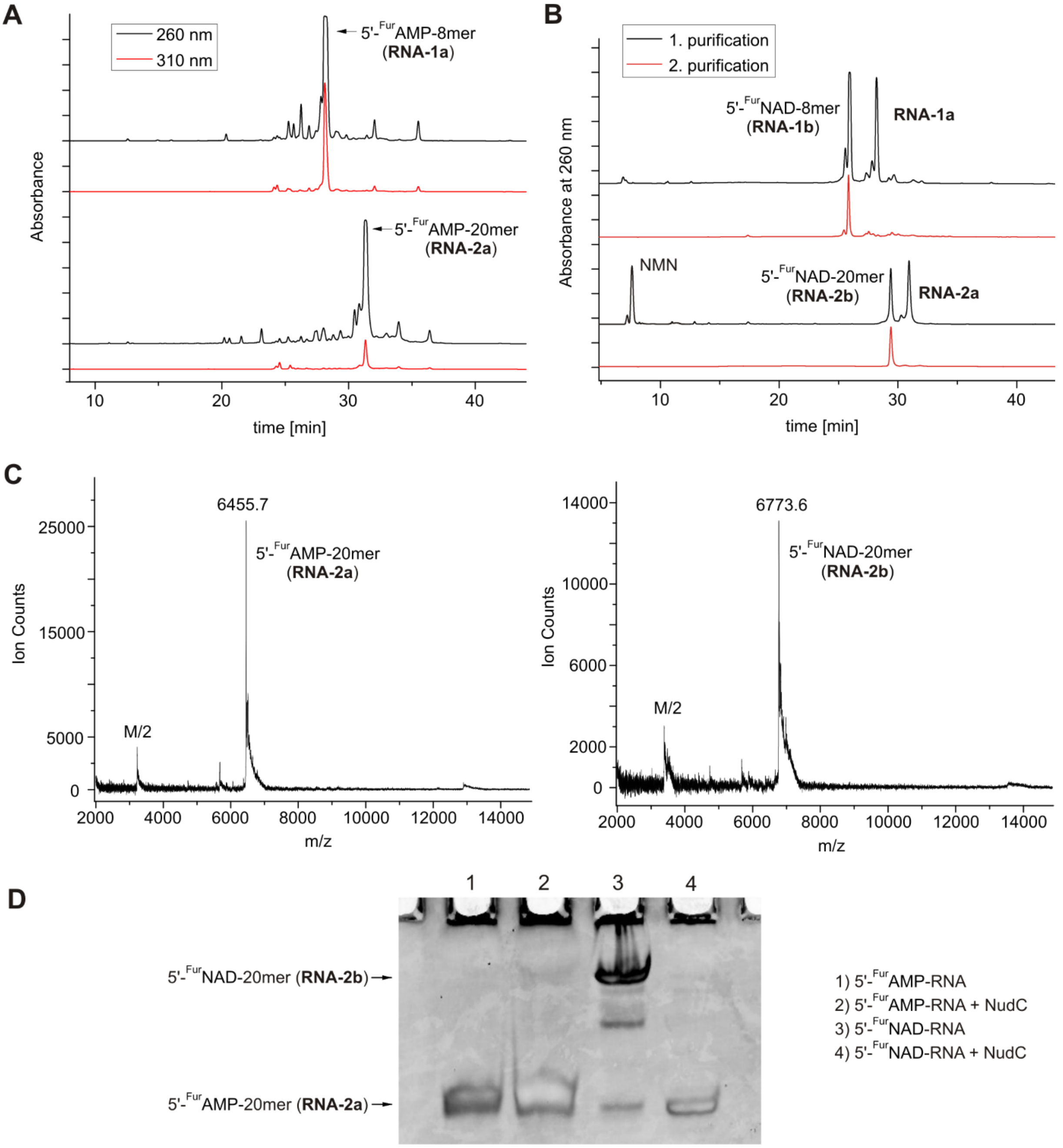

Figure S6: Purification and characterisation of synthesized 20mer RNAs.

(A) HPLC purification of the chemically synthesized 5′-P-^{Fur}A-RNAs displayed for an 8mer (**RNA-1a**) and a 20mer (**RNA-2a**). The 8mers (**RNA-1a/3a/4a**, 50 – 75 %) and 20mer (**RNA-2a**, 35 %) were obtained in moderate yields. Absorbance at 310 nm indicates oligonucleotides bearing the 8-(furan-2-yl)-adenosine modification. (B) HPLC purification of synthesized 5′-^{Fur}NAD-RNAs (**RNA-1b**, **RNA-2b**) with a Luna 5 µm C18(2) column (100 Å (250 x 15 mm), Phenomenex) at a flow rate of 5 ml/min, 5 – 20 % buffer B in 35 min). In most cases two successive purification runs were necessary. (C) MALDI-MS of 5′-^{Fur}AMP-20mer (**RNA-2a**) and 5′-^{Fur}NAD-20mer (**RNA-2b**); *m/z* calculated for (M+H): 6451.8; found 6455.7 and (M) 6767.9; found 6773.6. (D) 18% -APB-gel analysis of HPLC-purified (as in panel B) 5′-^{Fur}NAD-20mer (**RNA-2b**) and 5′-^{Fur}AMP-20mer (**RNA-2a**) generated by NudC digest of **RNA-2b**. The middle band in lane 3 is likely a degradation product that could not be removed by HPLC.

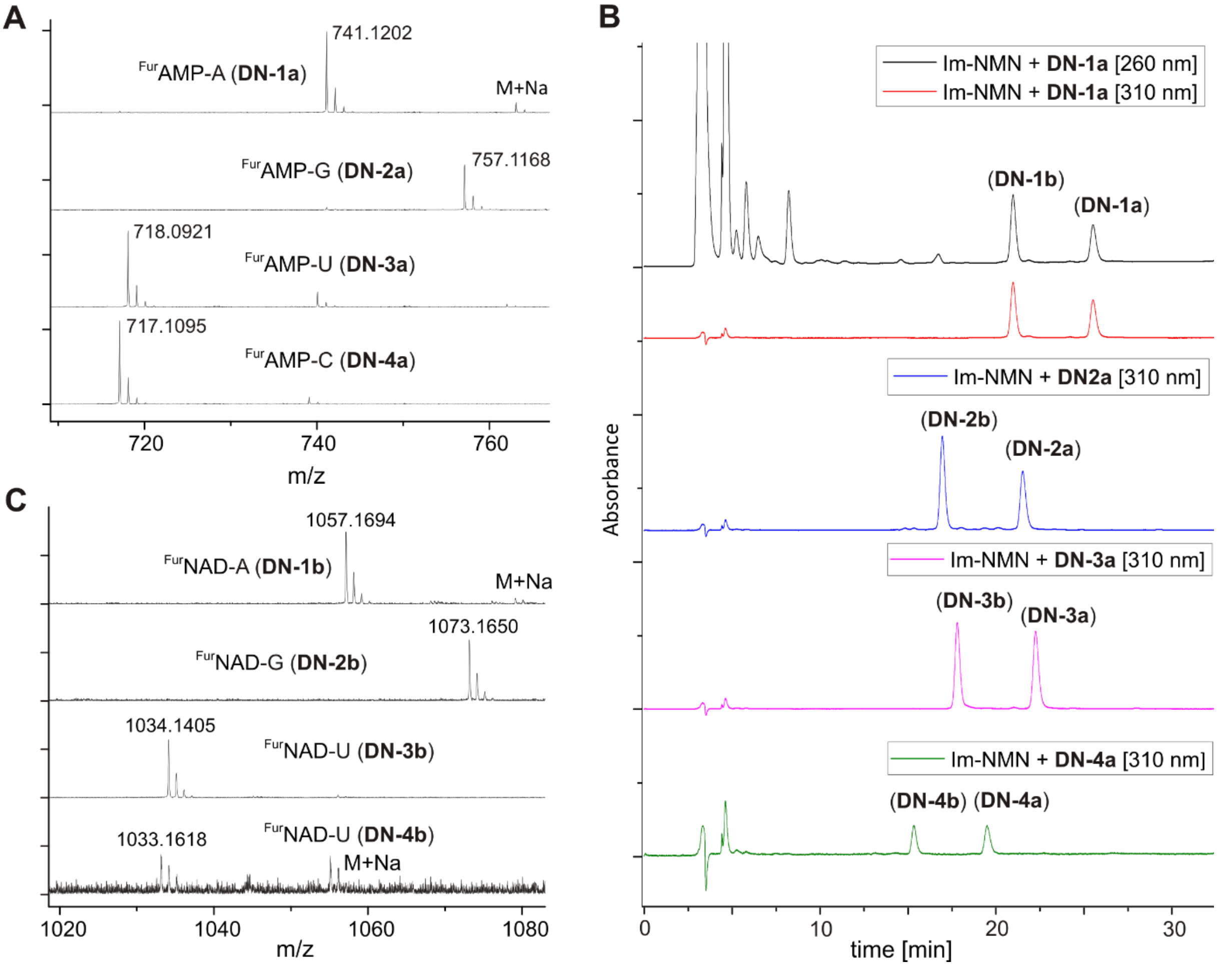

Figure S7: HRMS (ESI-TOF, negative) and HPLC analysis and identification of synthesised dinucleotides (**DN-1a – DN-4a** and **DN-1b – DN-4b**).

The yields of obtained dinucleotides (**DN1a – DN4a,**see Supplementary Table S2, 80 – 95 %) were high as expected. (A) HRMS (ESI-TOF, neg.) of HPLC purified dinucleotides (^{Fur}AMP-N) **DN-1a – DN-4a,** calculated for $(M\text{-}H^+)^-$: **DN-1a**: cal. 741.1189, found 741.1202; **DN-2a**: cal. 757.1138, found 757.1168; **DN-3a**: cal. 718.0917, found 718.0921; **DN-4a**: cal. 717.1077, found 717.1095. (B) Analytical HPLC of crude reaction mixtures after 5′-NAD generation of dinucleotides (^{Fur}AMP-N) using Im-NMN and yielding ^{Fur}NADpN (**DN-1b – DN-4b**) with 45 – 65 % conversion (absorbance at 310 nm). The formation of by-products was not observed. First line (absorbance at 260 nm) showing NMN and other decomposition products arising from Im-NMN. HPLC conditions: Phenomenex Luna 5 µm C18(2) 100 Å (250 x 4.60 mm), 5 - 15 % buffer B in 30 min, 1 ml/min.

(C) HRMS (ESI negative) of HPLC purified 5′-^{Fur}NAD dinucleotides (^{Fur}NADpN) **DN-1b – DN-4b,** calculated for $(M^+\text{-}2H^+)^-$: **DN-1b**: cal. 1057.1649, found 1057.1694; **DN-2b**: cal. 1073.1598, found 1073.1650; **DN-3b**: cal. 1034.1377, found 1034.1405; **DN-4b**: cal. 1033.1537, found 1033.1618.

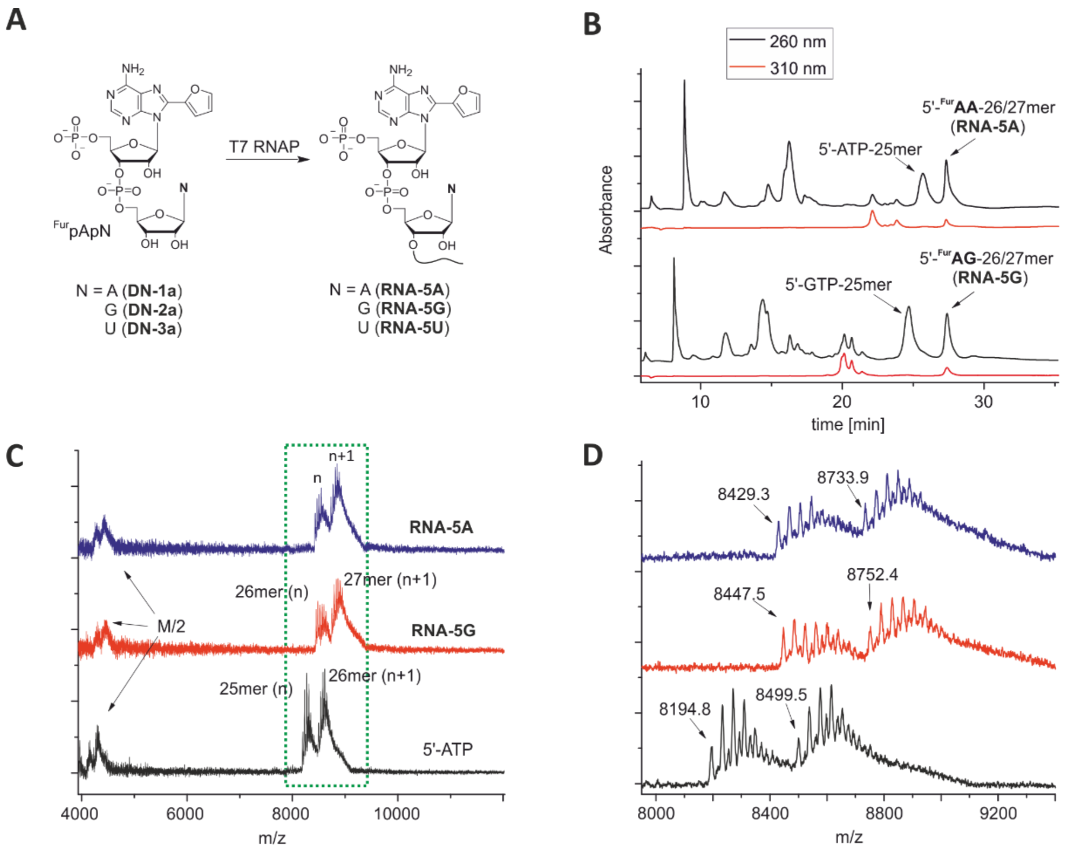

E

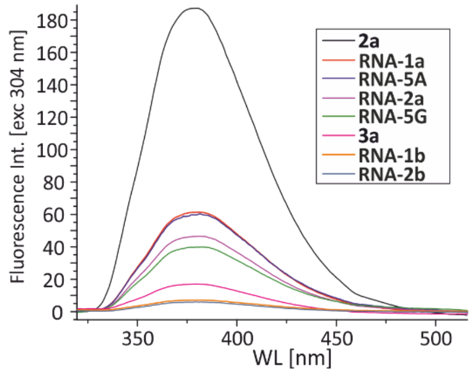

	Fluorescence Int. rel. (abs.)	Fluorescence Int. turn-on	Sequence
**2a**<br>**3a**	30.1 (187)<br>3 (18.6)	10	**FurA**<br>**FurNAD**
**RNA-1a**<br>**RNA-1b**	10 (62)<br>1.1 (6.7)	9.3	**FurA**-CAGUAUU<br>**FurNAD**-CAGUAUU
**RNA-2a**<br>**RNA-2b**	7.5 (46.5)<br>1 (6.2)	7.5	**FurA**-CAUUAUUUGGUAUGCCCGU<br>**FurNAD**-CAUUAUUUGGUAUGCCCGU
**RNA-3a**<br>**RNA-3b**	12.2 (76)<br>1.3 (8)	9.5	**FurA**-*Rsp*-CAGUAUU<br>**FurNAD**-*Rsp*-CAGUAUU
**RNA-5A**<br>**RNA-5G**	9.7 (60.2)<br>6.5 (39.8)	--	**FurA**-ACAGCTCAGCCTACGAGCCTGAGCC<br>**FurA**-GACGCTCAGCCTACGAGCCTGAGCC

Figure S8: Enzymatic FurAMP-RNA synthesis, purification and analysis. Synthesis of 5′-8-(furan-2-yl)-AMP 26/27mer RNAs via IVT using initiator dinucleotides. (A) Incorporation of dinucleotides (**DN-1a – DN-3a**) by T7 RNA polymerase. (B) HPLC purification using a Phenomenex Luna 5 µm C18(2) 100 Å (250 x 4.60 mm), 5 - 15 % buffer B in 40 min, 1 ml/min. (C) Identification of obtained oligonucleotides FurAMP-26/27mer RNAs (n/n+1) (**RNA-5A, RNA-5G**) via MALDI-MS. (D) Zoom-in in green highlighted box shown in panel (C). Calculated masses *m/z* $(M+H)^+$: 5′-ATP-25mer (8199.8), RNA-5A-26mer (8425.1), RNA-5G-26mer (8440.9). Calculated mass *m/z* $(M+H)^+$ for additional pyrimidine incorporation (n+1) 304 m/z. (E) Comparison of fluorescence spectra of synthesized compounds bearing the 8-(furan-2-yl)-adenosine modification. **RNA-3a** and **RNA-3b** carry an abasic site (-Rsp-) in order to characterise the quenching effect of the downstream nucleobase.

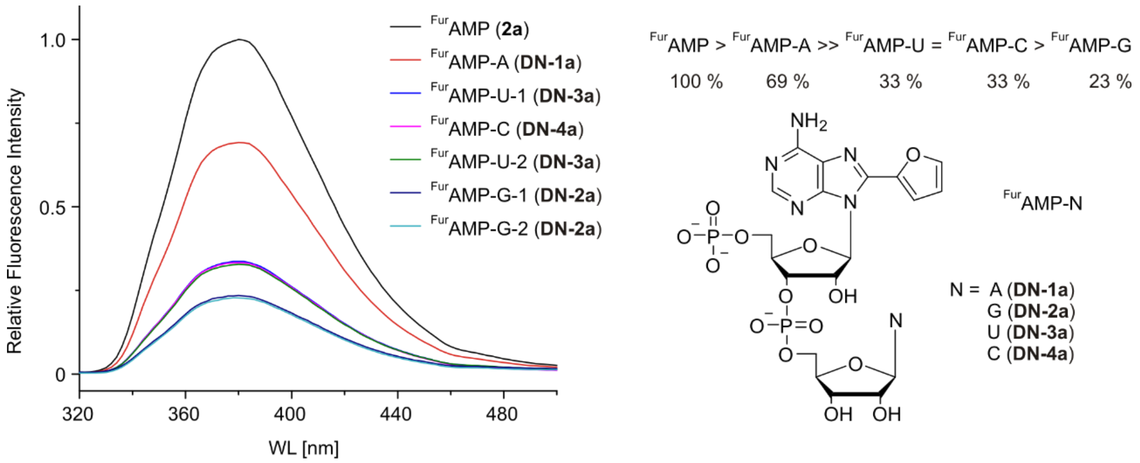

Figure S9: Impact of the neighbouring base on FurAMP fluorescence. Fluorescence spectra of dinucleotides **DN-1a – DN-4a** in comparison to 8-(furan-2-yl)-AMP (**2a**). Replicates for FurAMP-U and FurAMP-G are depicted as -1 or -2. After incorporation into a longer oligonucleotide these differences are reduced (Figure S8 E).

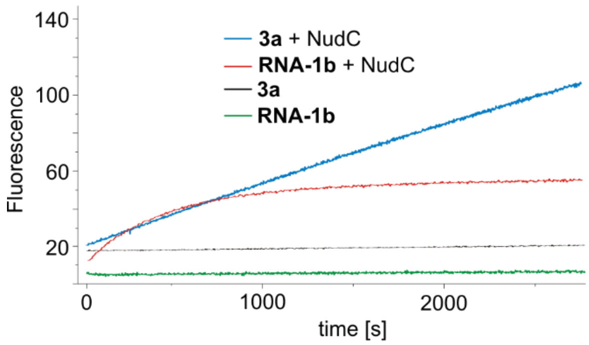

Figure S10: Fluorescence time-course measurement using 8-(furan-2-yl)-NAD (**3a**, 4.5 µM) and 8-(furan-2-yl)-NAD-RNA (**RNA-1b**, 4.5 µM) in the presence of NudC (4 µM). In comparison to Figure 4B where the fluorescence turn-on is depicted, absolute fluorescence intensities are shown here.

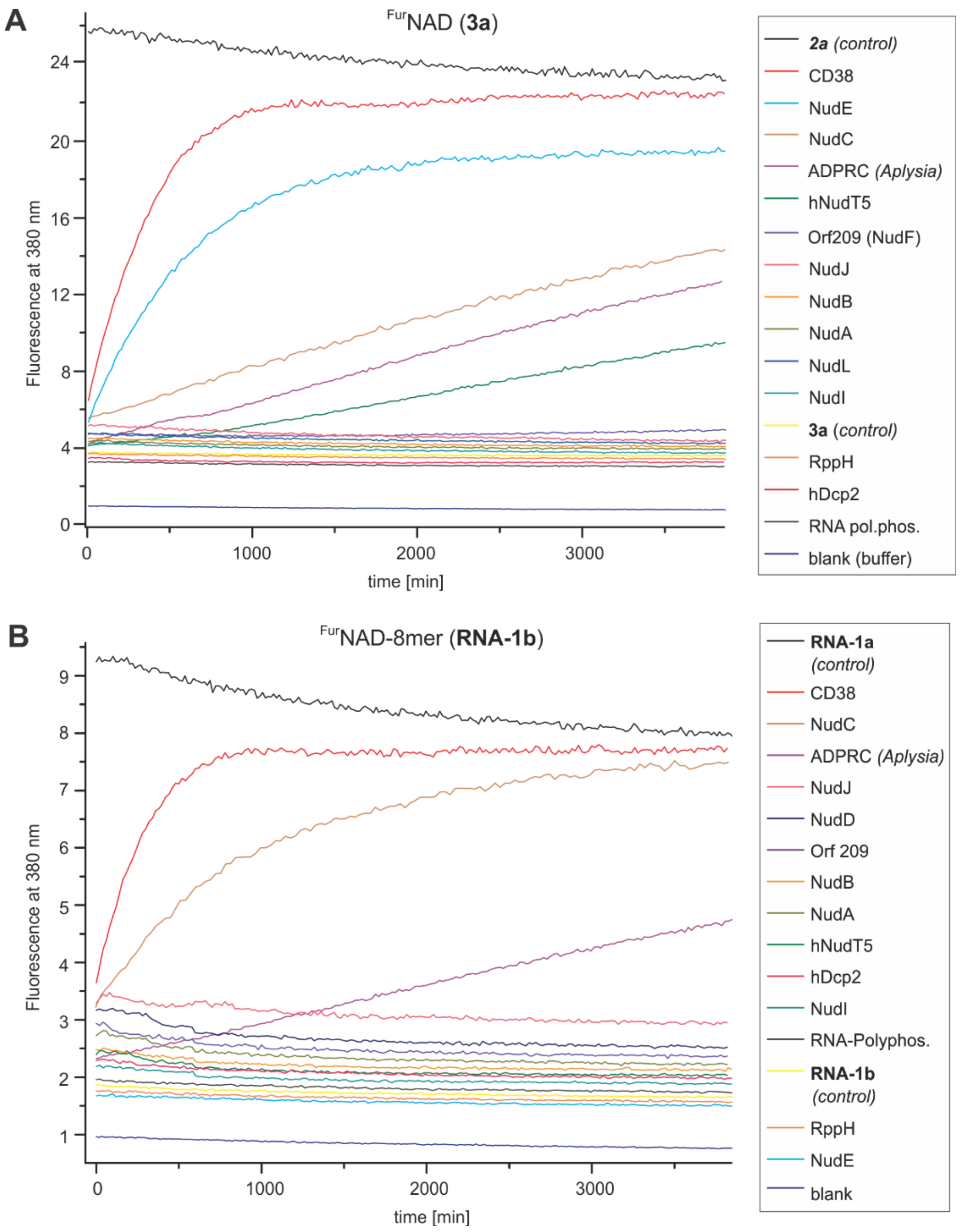

Figure S11: Screening of enzymes that hydrolyse FurNAD or remove the NAD-cap. Fluorescence measurements were performed in real time using a fluorescence microplate reader. Applied substrates: FurNAD (**3a**) (A) and FurNAD-8mer (**RNA-1b**) (B), each 4.5 µM, show a fluorescence turn-on (exc 304 nm/em 380 nm) in presence of active enzymes. Controls **3a** (I = 4) and **2a** (I = 24) and **RNA-1b** (I = 2) and **RNA-1a** (I = 9) while blank (buffer, I = 1) still has to be subtracted, resulting in an up to 8-fold fluorescence turn-on. I = 1 corresponds to an intensity of 1000 (AU) at the Tecan Safire2 well plate reader.

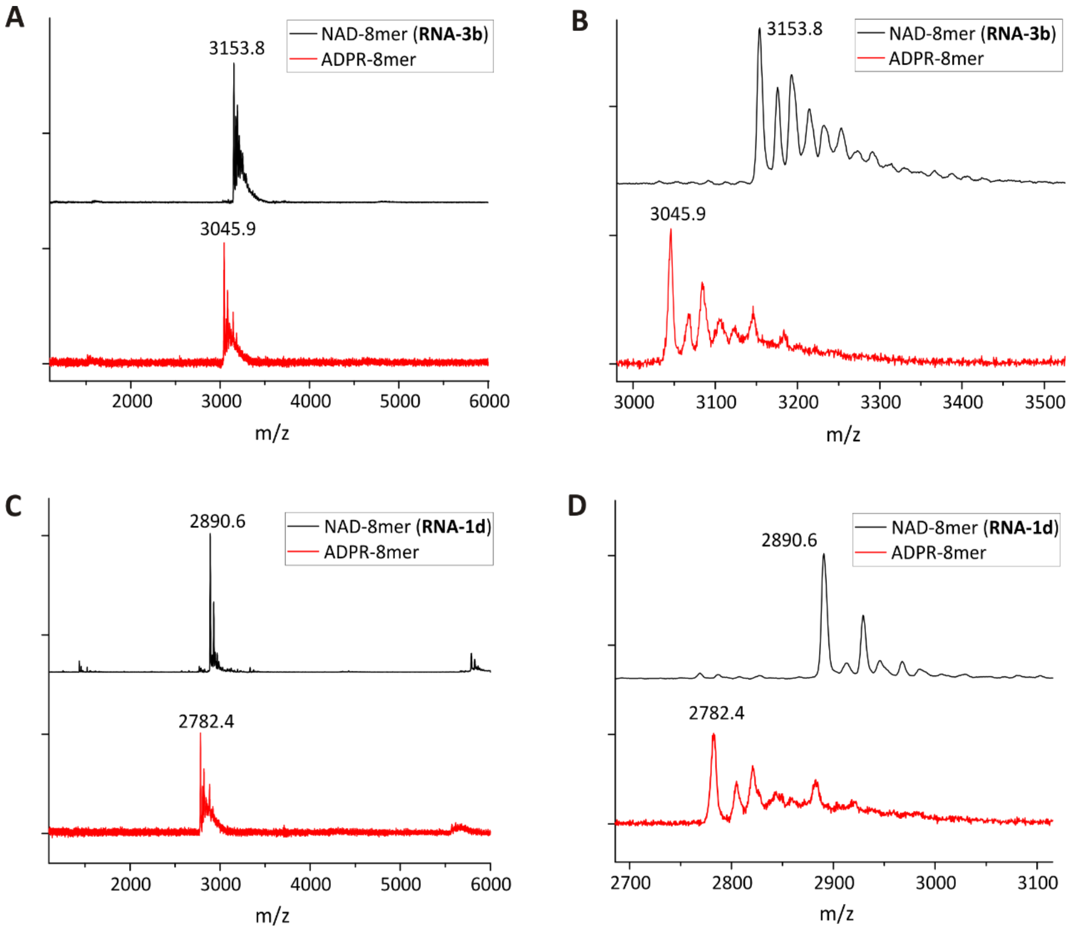

Figure S12: Identification of CD38 reaction products by MALDI-MS. Mass spectra of obtained 5′-ADPR-RNAs originating from 5′-NAD-RNAs after hydrolysis by CD38. (A, B) 5′-^{Fur}NAD-Rib$_{sp}$-RNA (**RNA-3b**) and (C, D) NAD-8mer (**RNA-1d**) without a ^{Fur}A modification (*black*). Respective ADPR-RNAs (*red*): calculated for (B) $C_{90}H_{116}N_{29}O_{71}P_{10}^{+}$, 3048.37 and (D) $C_{81}H_{105}N_{29}O_{64}P_{9}^{+}$ 2786.35 m/z. (calculated mass difference [NAD - ADPR] = [-Nicotinamide +OH]: 108.062 m/z)

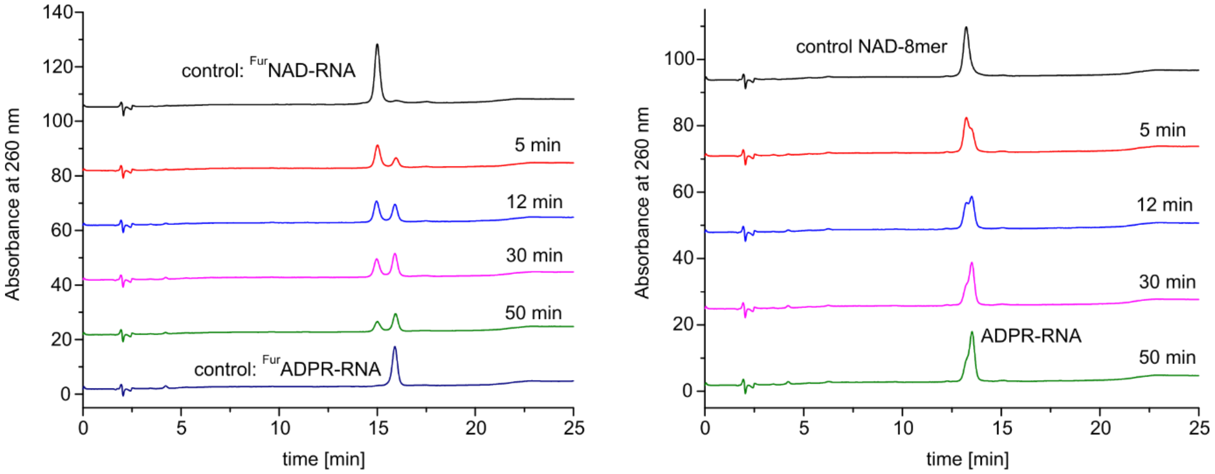

Figure S13: HPLC analysis of the hydrolysis of NAD-8mer and FurNAD-8mer by CD38. Comparison of CD38 (1 nM) activity on FurNAD-8mer (**RNA-1b**, 4.5 µM) and NAD-8mer (**RNA-1d**, 4.5 µM). No major influence of the 2-furanyl substituent in **RNA-1b** is visible.

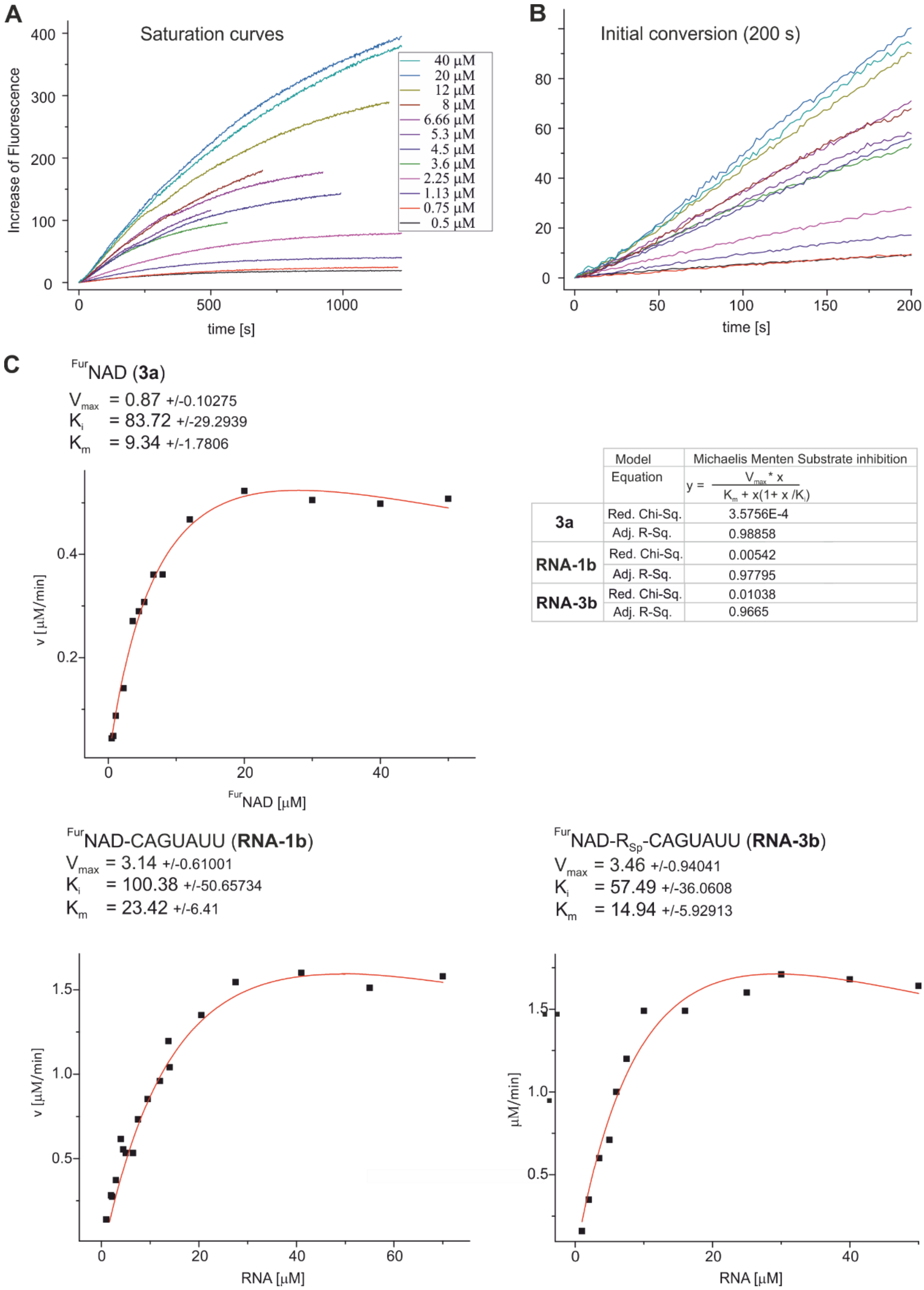

Figure S14: Determination of the kinetic parameters of CD38 (2.5 nM) in the presence of different concentrations of FurNAD and FurNAD-RNAs. (A) Initial rates of conversion of FurNAD (**3a**) were measured applying linear fit calculations (subtracted initial emission intensities at t_0) within the first 200 seconds. (B) Zoomed-in initial phase of experiments shown in panel (A). (C) Plots of the initial rates vs. substrate concentration for the conversion of FurNAD (**3a**) and FurNAD-8mers **RNA-1b, RNA-3b** and fit of the data to a Michaelis-Menten scheme with substrate inhibition. **RNA-3b** carries an abasic site (-Rsp-) in order to characterize the quenching effect of the downstream nucleobase.

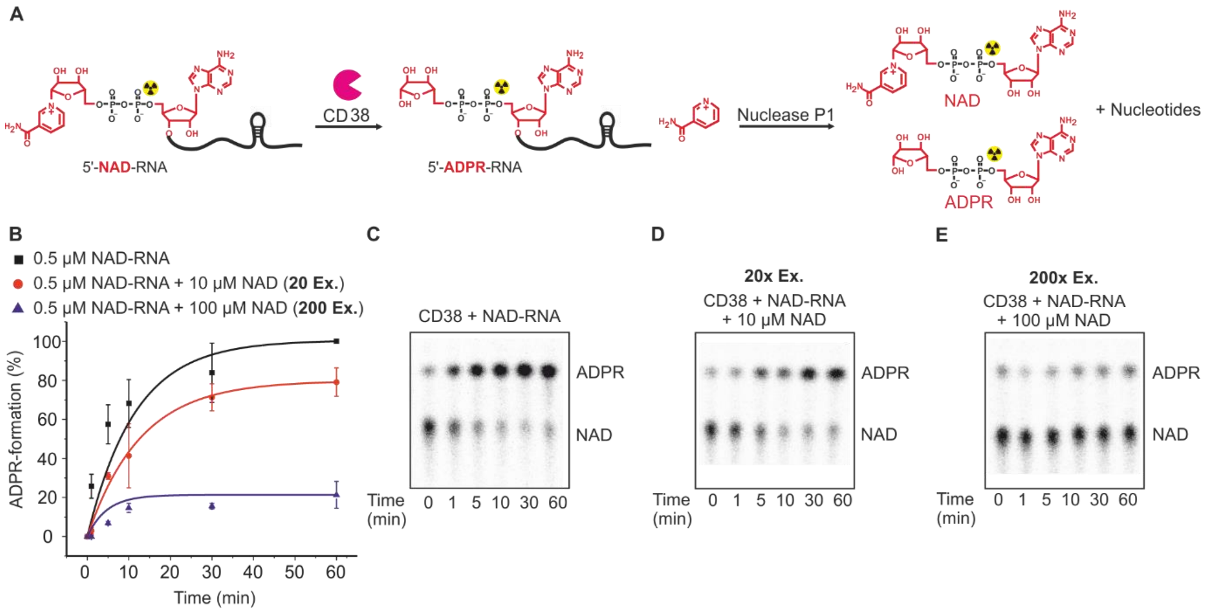

Figure S15: Validation of CD38 activity and specificity using radioactive 5′-NAD-capped RNA. (A) Schematic representation of activity of CD38 on NAD-capped RNA and treatment with nuclease P1. (B) Enzyme kinetics of CD38 on radioactive 5′-NAD-capped RNA in the presence of a 20 and 200 fold excess of NAD over NAD-RNA. (C-E) TLC analysis of enzyme kinetics shown in panel (B).

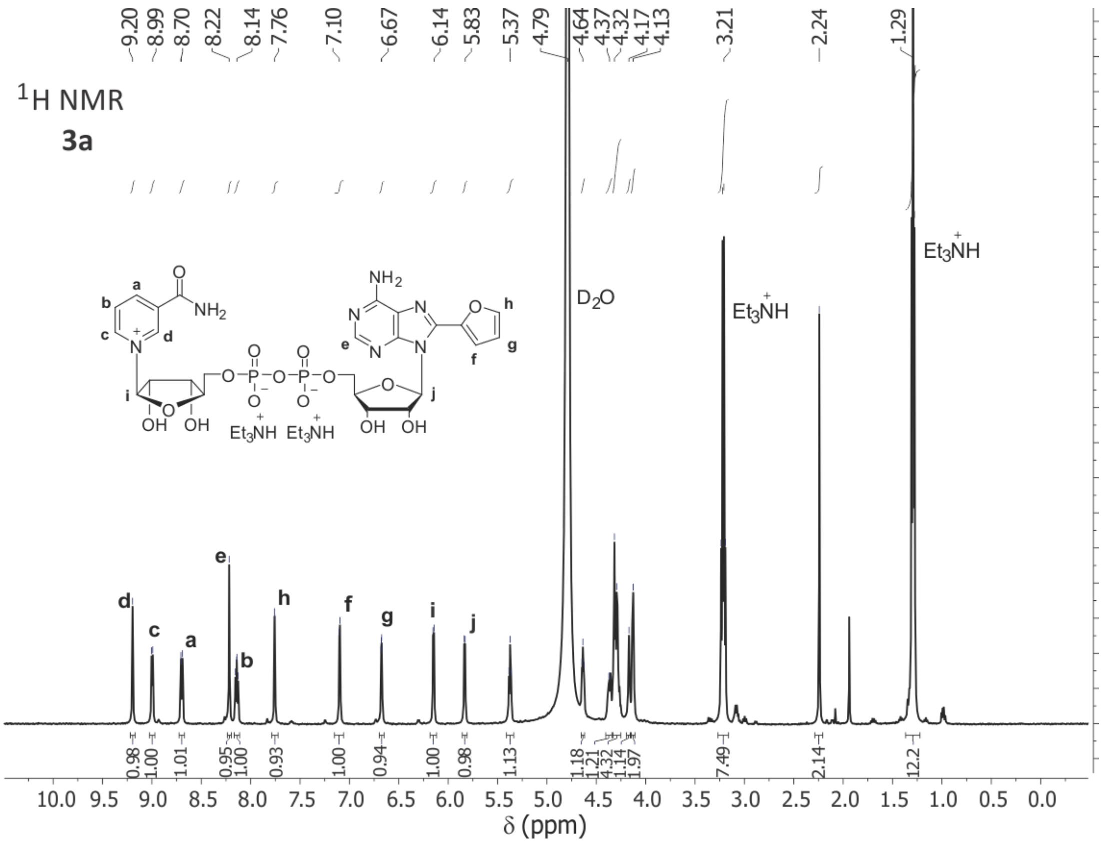

Figure S16: ^{1}H-NMR spectrum of **3a**.

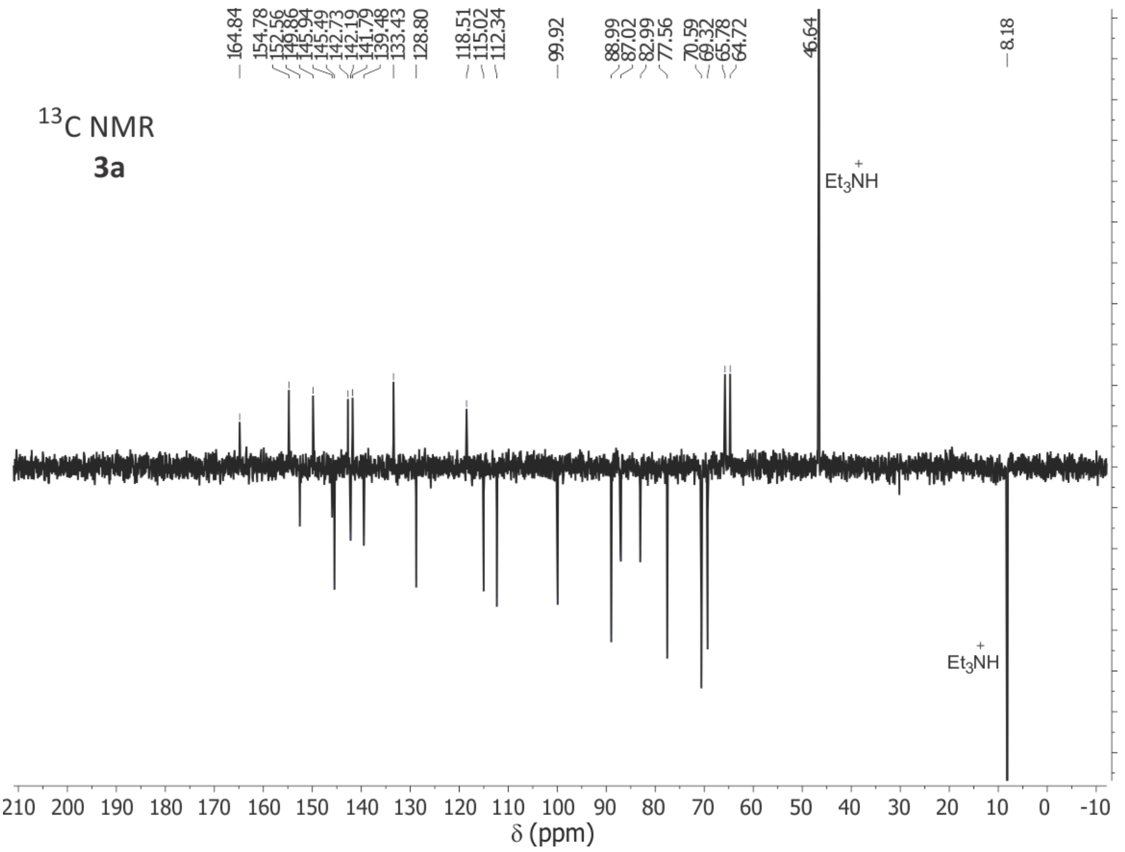

Figure S17: ^{13}C-NMR spectrum of **3a**.

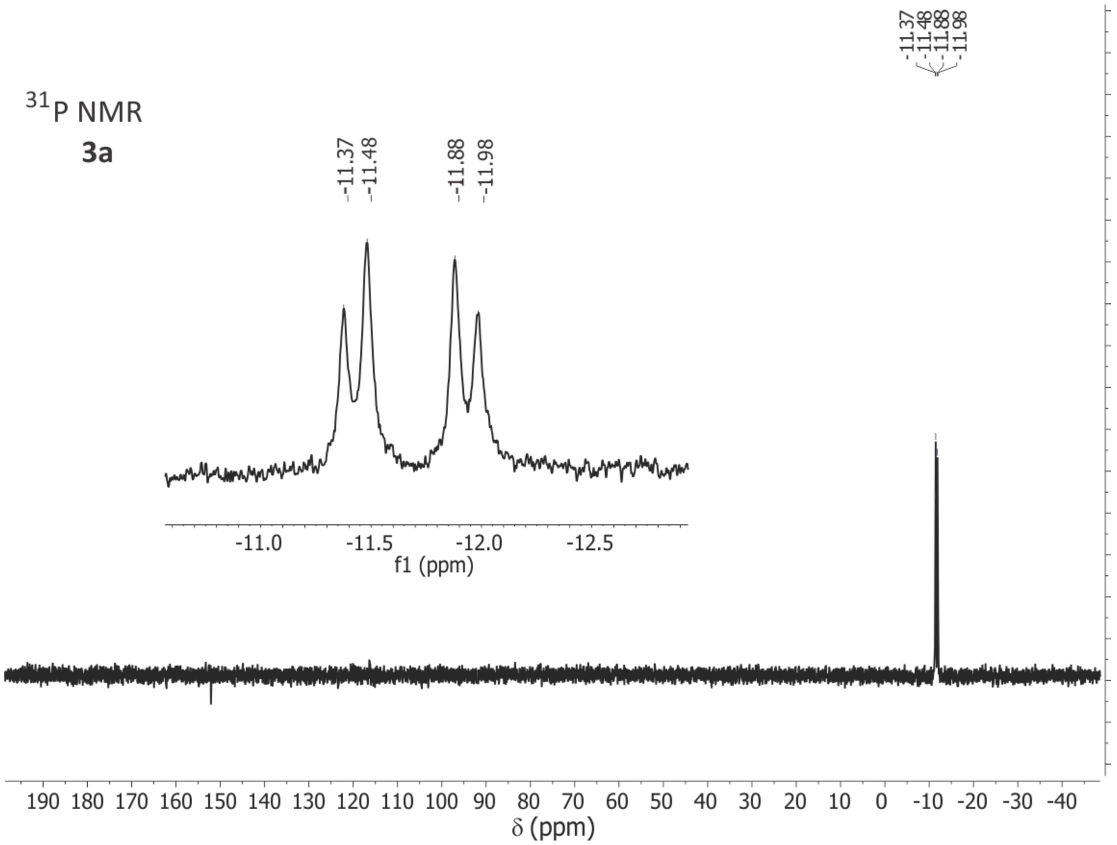

Figure S18: ^{31}P-NMR spectrum of **3a**.

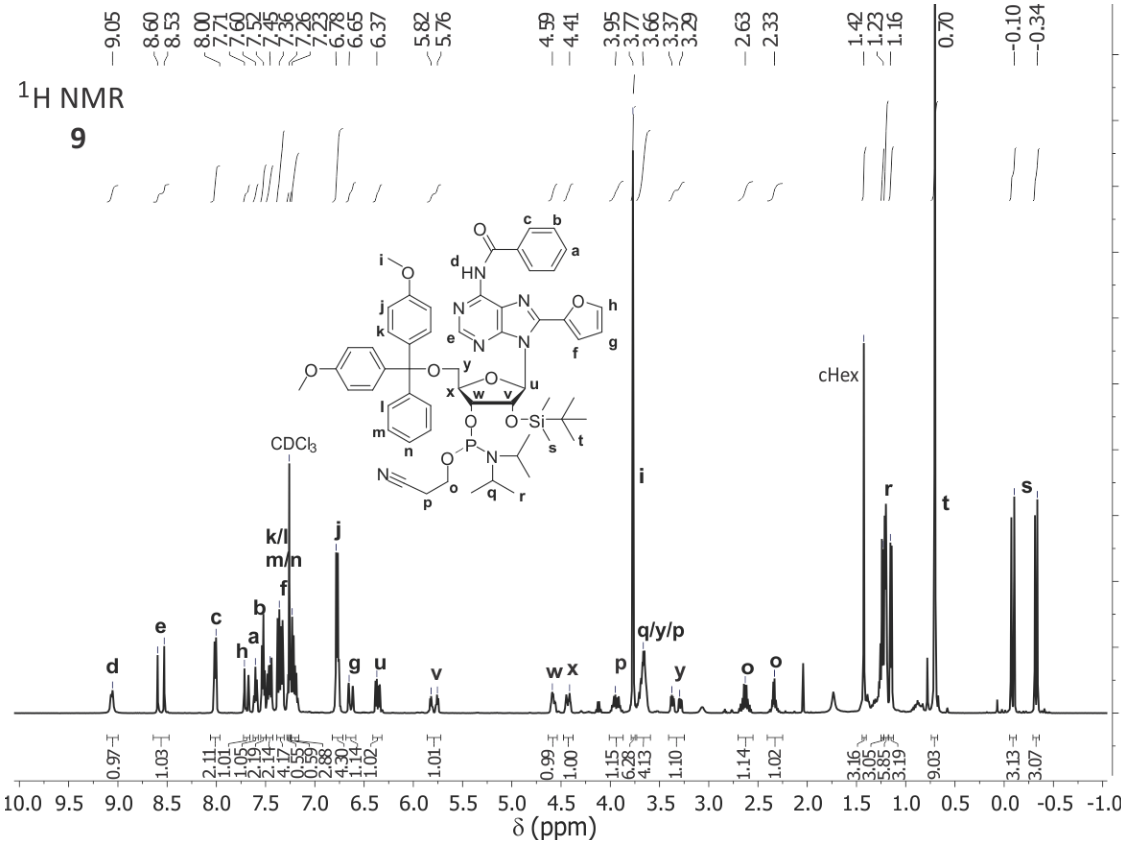

Figure S19: ^{1}H-NMR spectrum of the phosphoramidite **9**.

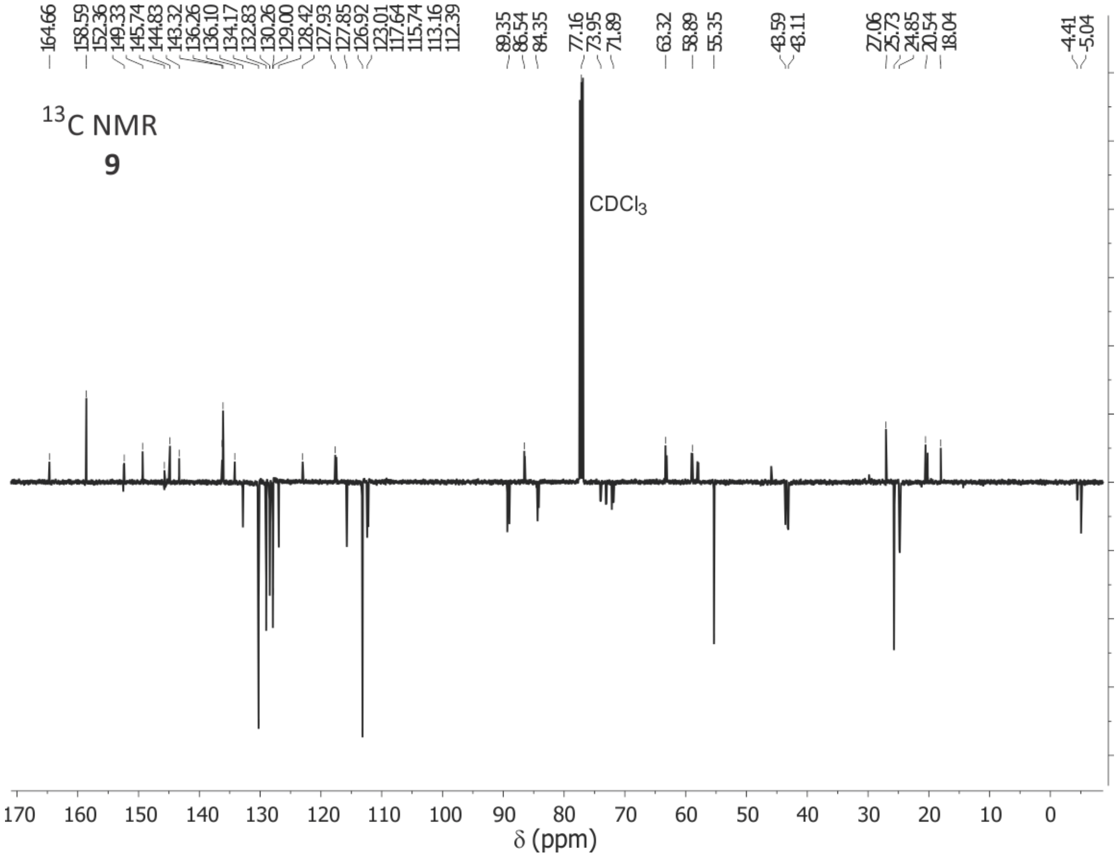

Figure S20: ^{13}C-NMR spectrum of the phosphoramidite **9**.

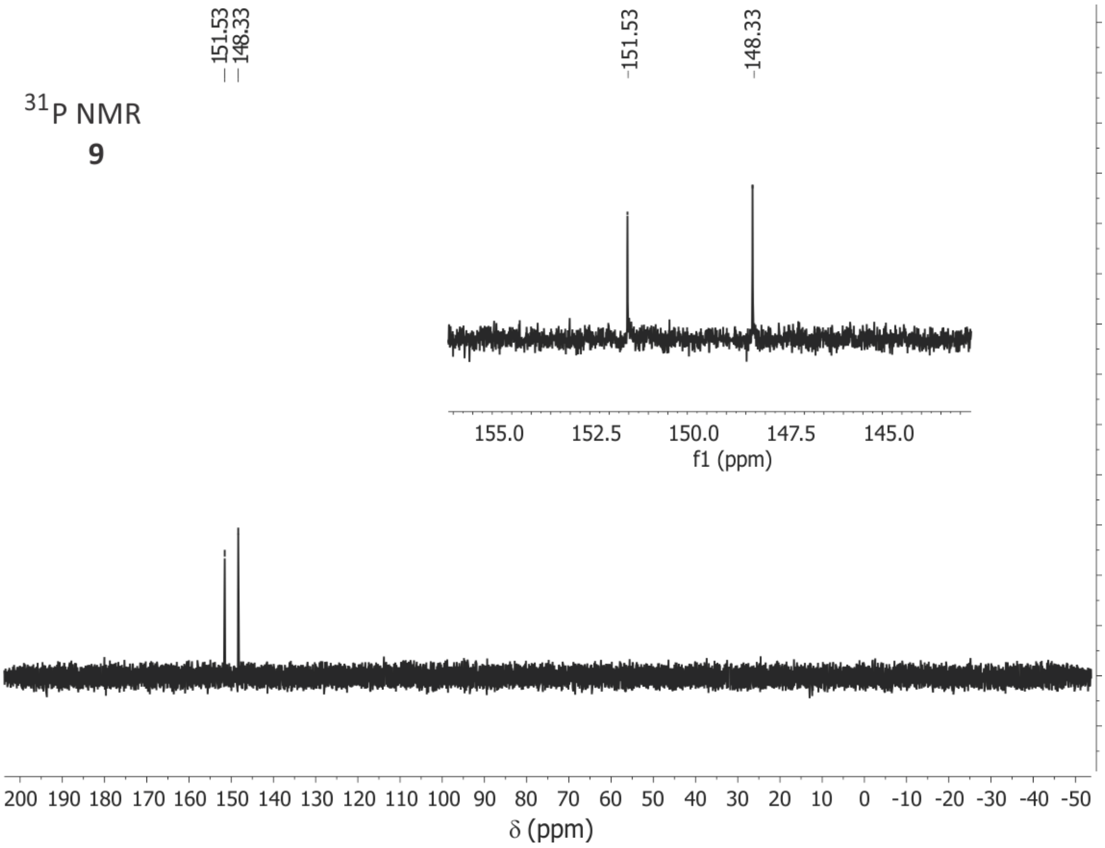

Figure S21: ^{31}P-NMR spectrum of the phosphoramidite **9**.

Chemical Synthesis

Suzuki-Miyaura reaction – synthesis of 8-Ar-AMP derivatives (2a – 2e)

Under an inert atmosphere (argon) **1** (1 eq), the boronic acid (1.5 eq), $Pd(OAc)_2$ 2 mol-%, TPPTS (6 mol-%) and K_2CO_3 were transferred to a Schlenk flask and degassed water (10 ml/mmol) was added. The reaction mixture was stirred at 80 °C for 2 h. Afterwards the mixture was adjusted to pH 7 with 1 % HCl-solution. The solvent was removed under reduced pressure and the remaining solid was subjected to reversed-phase automated flash chromatography (SiO_2-C18 (Telos), TEAB buffer (0.1 M), 2 – 20 % ACN).

Coupling of phosphorimidazolides – Synthesis of 3a and 3b.

Im-NMN and 2a or 2b (1 eq) were dissolved in DMF (4 ml/ 0.1 mmol) and dry $MgCl_2$ (10 eq) was added. The reaction mixture was stirred for 5 h at rt. DMF was removed under reduced pressure. The remaining solid was dissolved in a small amount of 0.1 M TEAB buffer and subjected to automated reversed-phase flash chromatography. Further HPLC purification provided the appropriate purity required for the application in fluorescence based assays (Phenomenex Luna C18(2), 5 µm, 100 Å, 250 x 21 mm, 6 – 20 % buffer B in 35 min, 6.5 ml/min). The applied buffer system was 100 mM triethylammonium acetate pH 7.0 (buffer A) and 100 mM triethylammonium acetate in 80 % acetonitrile (buffer B).

β-Nicotinamide mononucleotide (**NMN**) (Liu and Visscher, 1994)

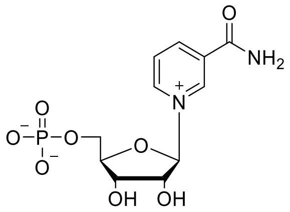

A solution of NAD (3.5 g, 5.28 mmol) and $ZrCl_4$ (6.15 g, 26.4 mmol) in 500 ml water was stirred at 50 °C for 30 min. The reaction was quenched with 245 ml of a 0.5 M solution of Na_3PO_4. After adjustment to pH 7 using HCl (2 M), a white precipitate formed. The suspension was centrifuged (8 min, 1000 rpm) the supernatant was collected and the pellet was washed two times with 200 ml water. The combined supernatants were concentrated to 1/4 of the original volume on a rotary evaporator. The remaining solution was purified with a column filled with Dowex 50WX8 (100-200 mesh, H^+-form, column-material: 2.5 x 30 cm). The resin was loaded by passing 1.5 l HCl (5 %) through a larger glass column equipped with the resin. Afterwards the resin was brought pH 5 by passing 1.5 l water until the flow-through reached pH 5. The resin was transferred to the 2.5 x 30 cm column and excess water was removed. 100 ml of the concentrated NMN/AMP solution was loaded on the ion exchange column, which was connected to the automated flash device and eluted with Millipore water (7 ml/min for 4 h). Initial fractions containing **NMN** eluted from the column as the free acid (80 – 180 min). A colourless solid **NMN** (**1**) (978 mg, 2.92 mmol, 55 %) was obtained after evaporation of the solvent. Following fractions contained AMP.

$\mathbf{R_f}$ = 0.25 (**NMN**) (SiO_2, EtOH/ NH_4OAc (1 M) [7:3]); $\mathbf{R_f}$ = 0,4 (NAD), 0.7 (AMP)

^{1}H-NMR (500 MHz, D_2O) δ [*ppm*] 9.49 (s, 1H), 9.31 (d, J = 6.3 Hz, 1H), 9.03 – 8.99 (m, 1H), 8.32 (dd, J = 8.0, 6.3 Hz, 1H), 6.24 (d, J = 5.5 Hz, 1H), 4.68 – 4.65 (m, 1H), 4.59 (d, J = 5.2 Hz, 1H), 4.47 (dd, J = 5.2, 2.6 Hz, 1H), 4.36 – 4.31 (m, 1H), 4.20 – 4.15 (m, 1H). **^{13}C-NMR** (75 MHz, D_2O) δ [*ppm*] 165.50 (q, CO), 145.65 (CH), 142.15 (CH), 139.53 (CH), 133.62 (q), 128.19 (CH), 99.65 (CH, C-5′), 87.12(CH, d, C-4′), 77.42(CH, C-3′), 70.71 (CH, C-4′), 63.86 (CH_2, d, C-5′). **^{31}P-NMR** (202 MHz, D_2O) δ [*ppm*] -0.03

β-Nicotinamide ribose-5′-phosphorimidazolide (**Im-NMN**) (Höfer et al., 2016)

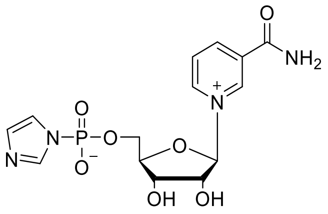

NMN (**1**) (100 mg, 299 µmol) was co-evaporated with 5 ml dry DMF. Afterwards, 500 mg 1,1′-carbonyldiimidazole (CDI) and 12 ml dry DMF were added and the mixture was stirred at rt overnight. ^{31}P-NMR analysis of 0.2 ml reaction solution mixed with 0.2 ml DMSO-d_6 (-10.9 ppm), was indicating that the intermediate 2′-3′-carbonate was formed. After addition of 20 ml 0.2 M TEAB buffer the reaction solution was stirred for 5 h at rt, which led to the hydrolysis of the carbonate and formation of **Im-NMN** (-10.5 ppm). The solvent was removed under reduced pressure at rt and the remaining solid was dissolved in 9 ml DMF. Within 10 min at room temperature a precipitate was formed and removed by filtration. **Im-NMN** was precipitated by addition of 80 ml acetone containing 1.4 g $NaOCl_4$. After centrifuging (8 min, 3000 rpm, rt) the pellet was washed with 45 ml acetone. The washing procedure was repeated 3 times. (Instead of centrifuging for concentration of **Im-NMN** it can also be filtered off and washed with acetone, which was preferred later) The solid was dried under reduced pressure and yielded 71 mg **Im-NMN** (**2**) (185 µmol, yield: 62 %).

^{1}H-NMR (500 MHz, DMSO-d_6) δ [*ppm*] 9.88 (s, 1H), 9.45 (s, 1H), 9.24 (d, J = 6.2 Hz, 1H), 9.06 – 9.03 (m, 1H), 8.30 (dd, J = 8.1, 6.2 Hz, 1H), 8.11 (s, 1H), 7.67 – 7.66 (m, 1H), 7.14 – 7.12 (m, 1H), 6.89 (s, 1H), 6.15 (d, J = 5.8 Hz, 1H), 6.03 (s (br), 1H, OH), 5.63 (s (br), 1H, OH), 4.34 – 4.30 (m, 1H), 4.29 – 4.25 (m, 1H), 4.09 – 4.02 (m, 2H), 3.98 – 3.91 (m, 1H). **^{13}C-NMR** (126 MHz, DMSO-d_6) δ [*ppm*] 162.43 (q, CO), 146.13 (CH), 143.30 (CH), 139.10 (CH, d), 134.14 (q), 128.64 (CH, d), 127.86, 119.83 (CH, d), 99.59 (CH, C-1′), 87.24 (CH, d, C-2′), 77.79 (CH, C-3′), 70.46 (CH, C-4′), 64.54 (CH_2, d, C-5′). **^{31}P-NMR** (202 MHz, DMSO-d_6) δ [*ppm*] -10.37.

HRMS (ESI-TOF positive) *m/z* calculated for $C_{14}H_{17}N_4O_7P$-Na^+ $[M-H^+ + Na^+]^+$ 407.0727, found 407.0726.

8-Bromo-adenosine-5′-monophosphate (**1**)

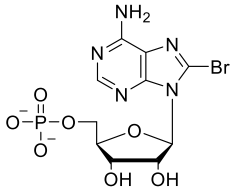

AMP (free acid) (2.30 g, 6.70 mmol, 1 eq) was dissolved in aqueous KH_2PO_4 buffer (0.25 M, pH 5, 460 ml). Afterwards bromine (2.40 ml, 47.0 mmol, 7 eq) was added. The mixture was stirred overnight at rt in the dark. The aqueous solution was washed three times with 300 ml DCM. The water was removed under reduced pressure on a rotary evaporator. The remaining solid was dissolved in 0.1 M TEAB buffer and purified via automated flash chromatography (SiO_2-C18 (Telos), 0.1 M TEAB buffer, 2 – 20 % ACN). The di-triethylammonium salt of 8-Br-AMP (**3**) was obtained as a red solid (610 mg, 971 µmol, 15 %). [29]

^{1}**H-NMR** (500 MHz, D_2O) δ [*ppm*] 8.18 (s, 1H, H-Ar), 6.10 (d, *J* = 6.1 Hz, 1H, H-1′), 5.31 (dd, *J* = 6.1 Hz, *J* = 6.0 Hz, 1H, H-2′), 4.60 (dd, *J* = 4.7, 6.0 Hz, 1H, H-3′), 4.27 (dt, *J* = 4.7, 11.1 Hz, 1H, H-4′), 4.07 (ddd, *J* = 5.9, 11.1, 17.4 Hz, 2H, H-5′). ^{13}**C-NMR** (126 MHz, D_2O) δ [*ppm*] 154.15 (q), 152.82 (CH), 150.22 (q), 128.12 (q), 119.15 (q), 89.20 (CH, C-1′), 83.82 (CH, d, C-2′), 70.61 (CH, C-3′), 69.68 (CH, C-4′), 63.76 (CH_2, C-5′).

HRMS (ESI-TOF, neg.) *m/z* calculated for $C_{10}H_{14}BrN_5O_7P^-$ $[M\text{-}H^+]^-$ 423.9663, found 423.9655.

8-(Furan-2-yl)-adenosine-5′-monophosphate (^{Fur}**AMP**, **2a**)

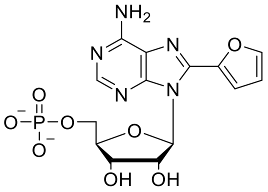

The synthesis was carried out according to the instructions in *Suzuki-Miyaura reaction*. Therefore **1** (120 mg, 191 µmol, 1 eq) and furan-2-boronic acid (32 mg, 289 µmol, 1.5 eq) were used. The di-triethylammonium salt of **2a** (55 mg, 89 µmol, 47 %) was obtained as a pale orange solid.

^{1}**H-NMR** (500 MHz, DMSO-d$_6$) δ [*ppm*] 8.17 (s, 1H, H^8-Ad), 8.00 (dd, J = 1.8, 0.7 Hz, 1H, H-Fur), 7.40 (s, 2H, NH$_2$), 7.11 (d, J = 3.5 Hz, 1H, H-Fur), 6.76 (dd, J = 3.5, 1.8 Hz, 1H, H-Fur), 6.03 (d, J = 5.6 Hz, 1H, H-1′), 5.30 (dd, J = 5.6 Hz, 1H, H-2′), 4.30 (dd, J = 5.6, 3.8 Hz, 1H, H-3′), 4.06 – 3.97 (m, 2H), 3.80 – 3.73 (m, 1H). ^{31}**P-NMR** (202 MHz, DMSO-d$_6$) δ [*ppm*] -0.43.

HRMS (ESI-TOF, negative) *m*/*z* calculated for $C_{14}H_{14}N_5O_8P^-$ $[M\text{-}H^-]^-$ 412.0688, found 412.0664. (Mass spectrum see Figure S2.

8-(Furan-2-yl)-NAD (^{Fur}**NAD**, **3a**)

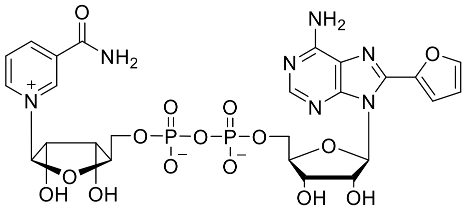

The synthesis was carried out according to the instructions in *coupling of phosphorimidazolides*. The di-triethylammonium salt of **2a** (89 mg, 145 µmol, 1 eq) was used. The di-triethylammonium salt of **3a** (42 mg, 45.0 µmol, 31 %) was obtained as a yellow solid after automated flash chromatography. Final HPLC purification provided pure **3a**.

^{1}**H-NMR** (500 MHz, D_2O) δ [*ppm*] 9.20 (s, 1H), 9.00 (d, *J* = 6.2 Hz, 1H), 8.70 (d, *J* = 7.8 Hz, 1H), 8.22 (s, 1H), 8.14 (dd, *J* = 7.8, 6.2 Hz, 1H), 7.76 (d, *J* = 1.9 Hz, 1H), 7.10 (d, *J* = 3.6 Hz, 1H), 6.67 (dd, *J* = 3.6, 1.8 Hz, 1H), 6.15 (d, *J* = 5.6 Hz, 1H, H-1'-NR.), 5.83 (d, *J* = 5.1 Hz, 1H, H-1'-Ad), 5.37 (dd, *J* = 6.0 Hz, 1H, H-2'-Ad), 4.63 (dd, *J* = 5.2 Hz, 1H, H-2'-NR), 4.41 – 4.24 (m, 6H), 4.20 – 4.10 (m, 3H). ^{13}**C-NMR** (126 MHz, D_2O) δ [*ppm*] 164.84 (q, CO), 154.78, 152.56, 149.86, 145.94, 145.49, 142.73, 142.19, 141.79, 139.48, 133.43, 128.80, 118.51, 115.02, 112.34, 99.92 (C-1', NR), 88.99 (C-1', Ad), 87.02 (C-2', NR), 82.99 (d, C-2' Ad), 77.56 (C-3', NR), 70.59 (C-4'), 69.32 (C-4'), 65.78 (C-5'), 64.72 (C-5'). ^{31}**P-NMR** (202 MHz, D_2O) δ [*ppm*] -11.43 (d, *J* = 21.0*)*, -11.94 (d, *J* = 21.0*)*. For NMR spectrum see Supplementary Figure S16 – S18. **HRMS** (ESI-TOF, neg.) *m*/*z* calculated for $C_{25}H_{28}N_7O_{15}P_2^-$ $[M-2H^+]^-$ 728.1124, found 728.1137. (Mass spectrum see Figure S2.

8-(Pyrrol-2-yl)-NAD (**PyrNAD**, **3b**)

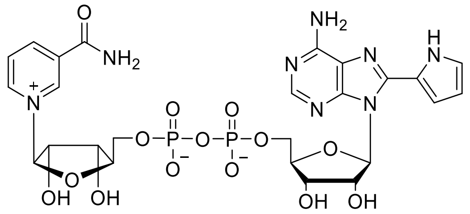

The synthesis was carried out according to the instructions in *coupling of phosphorimidazolides*. The di-triethylammonium salt of **2b** (75 mg, 122 µmol, 1 eq) was used. The di-triethylammonium salt of **3b** (32 mg, 34.3 µmol, 28 %) was obtained as a yellow solid after automated flash chromatography. HPLC purification provided pure **3b**.

^{1}H-NMR (500 MHz, D_2O) δ 9.16 (s, 1H), 8.99 (d, J = 6.3 Hz, 1H), 8.68 (d, J = 8.2 Hz, 1H), 8.22 – 8.21 (m, 1H), 8.16 – 8.11 (m, 1H), 7.20 – 7.17 (m, 1H), 6.73 – 6.69 (m, 1H), 6.39 – 6.36 (m, 1H), 6.01 – 5.98 (m, 1H), 5.80 (d, J = 4.8 Hz, 1H), 5.39 – 5.34 (m, 1H), 4.59 – 4.55 (m, 1H), 4.43 – 4.38 (m, 1H), 4.34 – 4.27 (m, 5H), 4.18 – 4.09 (m, 4H). **^{31}P-NMR** (202 MHz, D_2O) δ -11.42 (d, J = 21.1 Hz), -11.90 (d, J = 21.1 Hz). **HRMS** (ESI-TOF, neg.) *m/z* calculated for $C_{25}H_{29}N_8O_{14}P_2^-$ [M-2H$^+$]$^-$ 727.1284, found 727.1313. (Mass spectrum see Figure S2.)

8-Bromo-adenosine (**4**)

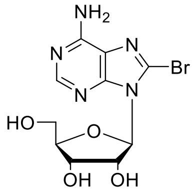

To an aqueous NaOAc buffer solution (pH 4, 22.5 ml, 0.5 M) adenosine (10 g, 37.4 mmol, 1 eq) was added. Afterwards a solution of bromine was added (408 µl Br_2 in 37.5 ml H_2O). The reaction was stirred for 20 h at rt in the dark. The excess bromine was neutralized with $Na_2S_2O_3$-solution (approx. 1 ml) until the mixture decolourized. The mixture was brought to pH 7 with 5 M NaOH-solution (approx. 5 ml). Cooling at 0 °C for 30 min resulted in precipitation of the product. The solid was washed with cold water and afterwards with cyclohexane. Residual solvent was removed under reduced pressure. **6** (8.71 g, 25.2 mmol, 67 %) was obtained as a light yellow solid.
^{1}**H-NMR** (300 MHz, DMSO-d_6) δ [*ppm*] 8.12 (s, 1H, H-Ar), 7.56 (s, 2H, NH_2), 5.83 (d, *J* = 6.6 Hz, 1H, H-1′), 5.51 (dd, *J* = 8.6, 4.0 Hz, 1H, OH), 5.46 (d, *J* = 6.4 Hz, 1H, OH), 5.23 (d, *J* = 4.6 Hz, 1H, OH), 5.13 – 5.05 (m, 1H, H-2′), 4.19 (ddd, *J* = 4.9, 4.6, 2.3 Hz, 1H, H-3′), 4.00 – 3.95 (m, 1H, H-4′), 3.73 – 3.47 (m, 2H, H-5′). **HRMS** (ESI-TOF, positive) *m*/*z* calculated for $C_{10}H_{12}BrN_5NO_4$-Na^+ $[M+Na^+]^+$ 367.9965, found 367.9958.

8-(Furan-2-yl)-adenosine (^{Fur}**A**, **5**)

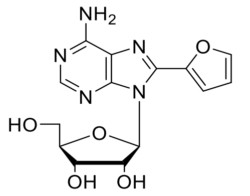

4 (3.00 g, 8.67 mmol, 1 eq), furan-2-boronic acid (1.64 g, 14.7 mmol, 1.7 eq), TPPTS (345 mg, 607 µmol, 0.07 eq), $Na_4[Pd(Cl_4]$ (76.5 mg, 260 µmol, 0.03 eq) and K_2CO_3 (1.80 g, 13 mmol, 1.5 eq) were stirred under an argon atmosphere in degassed water (160 ml) for 1.5 h at 80 °C. The mixture was brought to pH 7 with 2 M HCl. The solvent was removed under reduced pressure at a rotary evaporator. After column chromatography (DCM/MeOH 4:1; $\mathbf{R_f}$ = 0.48 (MeOH/DCM 1:5)) and removal of the solvent **5** (2.13 g, 6.40 mmol, 74 %) was obtained as a colourless solid.

^{1}**H-NMR** (300 MHz, DMSO-d_6) δ [*ppm*] 8.14 (s, 1H), 8.01 (dd, *J* = 1.8, 0.8 Hz, 1H), 7.56 (s, 2H (NH_2)), 7.14 (dd, *J* = 3.5, 0.8 Hz, 1H), 6.77 (dd, *J* = 3.5, 1.8 Hz, 1H), 6.11 (d, *J* = 6.7 Hz, 1H, H-1′), 5.75 (dd, *J* = 8.9, 3.5 Hz, 1H, OH), 5.44 (d, *J* = 6.5 Hz, 1H, OH), 5.19 (d, *J* = 4.7 Hz, 1H, OH), 5.16 – 5.08 (m, 1H, H-2′), 4.21 (ddd, *J* = 4.9, 4.7, 2.4 Hz, 1H, H-3′), 4.02 – 3.96 (m, 1H, H-4′), 3.75 – 3.49 (m, 2H, H-5′). **HRMS** (ESI-TOF, positive) *m*/*z* calculated for $C_{14}H_{16}N_5O_5^+$ [M+H$^+$] 334.1146, found 334.1142.

N[6]-Benzoyl-8-(furan-2-yl)-adenosine (**6**)

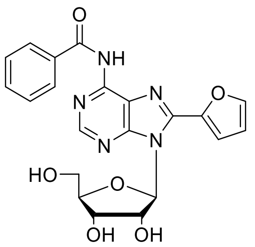

5 (2 g, 6.00 mmol, 1 eq) was co-evaporated with dry pyridine (5 ml). Under an argon atmosphere the residue was stirred in dry pyridine (100 ml) for 5 min. To the suspension TMSCl (3.80 ml, 30 mmol, 5 eq) was added and the mixture was stirred for 45 min at rt. After the addition of benzoyl chloride (3.49 ml, 30 mmol, 5 eq) the reaction proceeded for a further 2 h. To quench the mixture water (12 ml) and NH_3-aq. (25 ml, 25 %) were added and stirred for 30 min at rt. The mixture was extracted with DCM and washed with sat. $NaHCO_3$-solution. After drying over $NaSO_4$, the organic phase was filtered and evaporated under reduced pressure. The residue was co-evaporated three times with ethanol. After flash column chromatography (SiO_2, DCM – DCM/MeOH (19:1), TLC: **R**$_f$ = 0.66 (DCM/MeOH 20:3)) **6** was obtained as a colourless solid (2.12 g, 4.84 mmol, 81 %).

^{1}**H-NMR** (300 MHz, DMSO-d_6) δ [*ppm*] 11.28 (s, 1H (NH)), 8.77 (s, 1H), 8.10 – 8.03 (m, 3H), 7.70 – 7.62 (m, 1H), 7.60 – 7.53 (m, 2H), 7.30 (dd, *J* = 3.6, 0.8 Hz, 1H), 6.81 (dd, *J* = 3.6, 1.8 Hz, 1H), 6.22 (d, *J* = 6.2 Hz, 1H, H-1′), 5.51 (d, *J* = 6.1 Hz, 1H, H-OH), 5.36 – 5.28 (m, 1H, H-OH), 5.25 (d, *J* = 5.0 Hz, 1H, H-OH), 5.12 (ddd, *J* = 6.2, 5.0, 3.1 Hz, 1H, H-2′), 4.31 (ddd, *J* = 5.1, 5.0, 3.3 Hz, 1H, H-3′), 4.03 – 3.97 (m, 1H, H-4′), 3.79 – 3.53 (m, 2H, H-5′). **HRMS** (ESI-TOF, pos.) *m*/*z* calculated for $C_{21}H_{19}N_5O_6$ [M+H$^+$] 438.1408, found 438.1401.

N[6]-Benzoyl-5′-*O*-(4,4′-dimethoxytrityl)-8-(furan-2-yl)-adenosine (7)

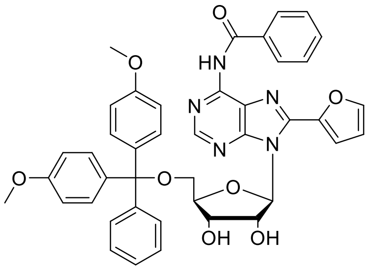

6 (1.83 g, 4.18 mmol, 1 eq) was co-evaporated with pyridine (5 ml). To the solution in dry pyridine (80 ml) DMTrCl (2.03 g, 5.99 mmol, 1.43 eq), DMAP (30 mg, 246 µmol, 0.3 eq) were added and stirred at 25 °C for 4 h under an argon atmosphere. Pyridine was removed under reduced pressure and the residue was further co-evaporated three times with ethanol and 1 % Et_3N. The remaining solid was further dried under high vacuum overnight. After flash column chromatography (SiO_2, DCM/Et_3N 99:1 – DCM/MeOH/Et_3N 95:4:1; TLC: $\mathbf{R_f}$ = 0.37 (DCM/MeOH 20:1, 1 % Et_3N)) **7** was obtained as a colourless solid (2.80 g, 3.79 mmol, 91 %). The di-/tri-protected by-products are eluting with DCM/Et_3N.

^{1}H NMR (300 MHz, $CDCl_3$) δ [*ppm*] 8.45 (s, 1H), 8.01 – 7.94 (m, 2H), 7.65 – 7.57 (m, 1H), 7.55 – 7.48 (m, 2H), 7.45 (dd, *J* = 1.8, 0.8 Hz, 1H), 7.40 – 7.34 (m, 2H), 7.30 (dd, *J* = 3.6, 0.8 Hz, 1H), 7.28 – 7.26 (m, 2H), 7.25 – 7.22 (m, 2H), 7.19 – 7.07 (m, 3H), 6.73 – 6.63 (m, 4H), 6.49 (dd, *J* = 3.6, 1.8 Hz, 1H), 6.33 (d, *J* = 4.4 Hz, 1H, H-1′), 5.77 (dd, *J* = 5.0 Hz, *J* = 4.4 Hz, 1H, H-2′), 4.82 (dd, *J* = 5.0 Hz, 1H, H-3′), 4.35 (dd, *J* = 4.8 Hz, 1H, H-4′), 3.71 (s, s; *J* = 4.5 Hz, 6H, OMe), 3.42 (ddd, *J* = 15.8, 10.2, 5.0 Hz, 2H, H-5′).

^{13}C NMR (126 MHz, $CDCl_3$) δ [*ppm*] 164.69 (q, 1C, C=O), 158.52 (q, 2C, C-OMe), 151.99 (q, 1C, C^2-Fur), 149.32 (p, 1C, C^2-Ad), 148.55 (q, 1C, C^6-Ad), 145.90 (CH, 1C, C^5-Fur), 145.71 (q, 1C, C^4-Ad), 144.98 (q, 1C, C^8-Ad), 142.60 (q, 1C, Bz), 136.16 (q, 2C, Bz), 133.47 (q, 1C, Bz), 132.90 (CH, 1C, DMTr), 130.26 (CH, 2C, DMTr), 130.09 (CH, 2C, Bz), 128.90 (CH, 2C, DMTr), 128.27 (CH, 4C, DMTr), 127.80 (CH, 2C, DMTr), 126.84 (CH, 1C, DMTr), 121.93 (q, 1C), 116.13 (CH, 1C, C^4-Fur), 113.09 (CH, 4C, DMTr), 112.42 (CH, 1C, C^3-Fur), 106.70 (CH, 1C,), 90.99 (CH, 1C, C-1′), 86.25 (q, 1C), 84.44 (CH, 1C, C-2′), 72.12 (CH, 1C, C-3′), 71.40 (CH, 1C, C-4′), 63.91 (CH_2, 1C, C-5′), 55.29 (CH_3, 2C, OMe). **HRMS** (ESI-TOF, pos.) *m/z* calculated for $C_{42}H_{37}N_5O_8$-Na^+ [M+Na^+] 762.2534, found 762.2552.

N^6-Benzoyl-2'-*O*-(*tert*-butyldimethylsilyl)-5'-*O*-(4,4'-dimethoxytrityl)-8-(furan-2-yl)-adenosin (**8**)

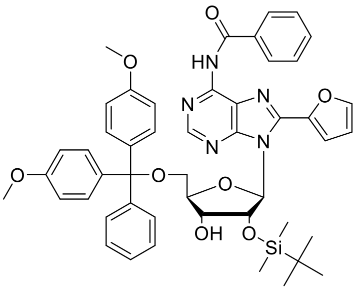

7 (1.9 g, 2.57 mmol, 1 eq) was co-evaporated with pyridine (5 ml). Under an argon atmosphere THF (26 ml), $AgNO_3$ (520 mg, 3.06 mmol, 1.2 eq) and pyridine (1 ml, 13.6 mmol, 5.3 eq) were added and stirred for 20 min at rt. After addition of TBDMSCl (500 mg, 3.32 mmol, 1.3 eq) stirring was continued for 4 h at 25 °C. The reaction mixture was extracted with ETAC and $NaHCO_3$-sol. (5 %) and dried over $NaSO_4$. The solvent was removed under reduced pressure. With flash column chromatography (SiO_2, cyclohexane/EtOAc/Et_3N 75:24:1; TLC: **Rf** = 0.34 (**8**), 0.23 (**8b**) (cHex/EtOAc – 1:1 + 1 % Et_3N)) the desired 2'-O-TBDMS protected product **8** eluted after the 2',3'-di-TBDMS protected and before the 3'-TBDMS protected compound **8b**. After removal of the solvent a colourless solid **8** was obtained (390 mg, 457 µmol, 18 %). The undesired 3'-TBDMS protected compound **8b** was the major by-product (659 mg, 772 µmol, 30 %), however, 50 % of the starting material (**8**) stayed unreacted and higher amounts TBDMSCl did not lead to an improvement.

2'-TBDMS (**8**) **^{1}H-NMR** (300 MHz, $CDCl_3$) δ [*ppm*] 9.06 (s, 1H), 8.58 (s, 1H), 8.08 – 7.98 (m, 2H), 7.70 (dd, *J* = 1.8, 0.8 Hz, 1H), 7.66 – 7.58 (m, 1H), 7.58 – 7.49 (m, 2H), 7.48 – 7.41 (m, 2H), 7.36 – 7.30 (m, 4H), 7.29 (dd, *J* = 3.5, 0.8 Hz, 1H), 7.25 – 7.10 (m, 2H), 6.80 – 6.71 (m, 4H), 6.64 (dd, *J* = 3.5, 1.8 Hz, 1H), 6.32 (d, *J* = 5.6 Hz, 1H, H-1'), 5.84 (dd, *J* = 5.6 Hz, 1H, H-2'), 4.55 – 4.49 (m, 1H, H-3'), 4.33 – 4.26 (m, 1H, H-4'), 3.77 (s, 6H, OMe), 3.46 (ddd, *J* = 46.5, 10.3, 5.1 Hz, 2H, H-5'), 0.78 (s, 9H), -0.06 (s, 3H), -0.31 (s, 3H). **HRMS** (ESI-TOF) *m/z* calculated for $C_{48}H_{52}N_5O_8Si^+$ [M+H$^+$] 854.3580, found 854.3561.

N^6-Benzoyl-2′-*O*-(*tert*-butyldimethylsilyl)-5′-*O*-(4,4′-dimethoxytrityl)-8-(furan-2-yl)-adenosyl-3′-(ß-cyanethoxy)-diisopropylaminophosphin (**9**)

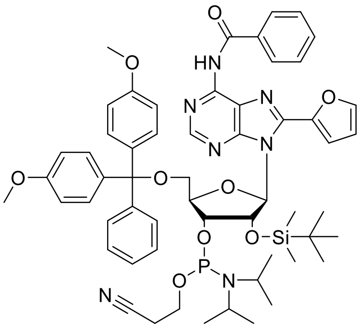

8 (145 mg, 170 µmol, 1 eq) was dried overnight under high vacuum. The solid foam was dissolved in THF (1.8 ml) containing *N*,*N*-diisopropylethylamine (200 µl)**.** After the addition of CEPCl (100 µL, 14 % Cl, 385 µmol, 2.27 eq) the clear solution was stirred for 1.5 h at rt. The cloudy mixture was subsequently subjected to flash column chromatography (SiO_2, cyclohexane/ETAC (1:1) + 1 % Et_3N; TLC: Rf = 0.55 (cHex/ETAC (1:1) + 1 % Et_3N)). The desired 3′-phosphoramidite **9** readily eluted without major impurities (151 mg, 144 µmol, 85 %).

^{1}H-NMR (500 MHz, $CDCl_3$) δ [*ppm*] 9.06 (s^I, s^J; 1H, NH), 8.56 (s^I, s^J; 1H), 8.04 – 7.98 (m, 2H), 7.69 (d^I, d^J; 1.8, 0.7 Hz, 1H), 7.63 – 7.57 (m, 1H), 7.55 – 7.50 (m, 2H), 7.49 – 7.42 (m, 2H), 7.39 – 7.31 (m, 5H), 7.28 – 7.25 (m, 2H), 7.25 – 7.15 (m, 3H), 6.80 – 6.73 (m, 4H), 6.63 (dd^I, dd^J; 3.5, 1.8 Hz, 1H), 6.36 (d^I, d^J; 6.8 Hz, 1H, H-1′), 5.79 (dd^I, dd^J, 6.8, 4.5 Hz, 1H, H-2′), 4.61 – 4.55 (m, 1H, H-3′), 4.47 – 4.38 (m, 1H, H-4′), 4.01 – 3.87 (m, 1H), 3.73 – 3.61 (m, 4H, H-5′ and others), 3.33 (dd^I, dd^J, *J* = 10.3, 4.7 Hz, 1H, H-5′), 2.69 – 2.57 (m, 1H), 2.40 – 2.28 (m, 1H), 1.23 (s^I, s^J; 3H, i-Pr), 1.20 (dd, *J* = 6.8, 3.7 Hz, 6H, i-Pr), 1.15 (s^I, s^J; 3H, i-Pr), 0.70 (s^I, s^J; 9H, Si-C(CH_3)$_3$), -0.09 (s^I, s^J; 3H, Si-CH_3), -0.32 (s^I, s^J; 3H, Si-CH_3). Two diastereomers (s^I, s^J - two singuletts. d^I, d^J - two doublets). **^{13}C-NMR** (126 MHz, $CDCl_3$) δ [*ppm*] 164.66, 158.59, 152.36, 149.33, 145.74, 144.83, 143.32, 136.26, 136.10, 134.17, 132.83, 130.26, 129.00, 128.42, 127.93, 127.85, 126.92, 123.01, 117.64, 115.74, 113.16, 112.39, 89.35, 86.54, 84.35, 77.16, 73.95, 71.89, 63.32, 58.89, 55.35, 43.59, 43.11, 27.06, 25.73, 24.85, 20.54, 18.04, -4.41, -5.04. **^{31}P-NMR** (202 MHz, $CDCl_3$) δ [*ppm*] 151.53, 148.33. Two diastereomers! NMR spectra see Supplementary Figure S19 – S21. **HRMS** (ESI-TOF, positive) *m*/*z* calculated for $C_{57}H_{68}N_7O_9PSi$-Na^+ [M+Na^+]$^+$ 1076.4478, found 1076.4438.

Supplementary Table S1. Sequences of RNAs synthesized by solid-phase oligonucleotide synthesis. Introduction of an abasic site into the RNA is depicted by -R_{Sp}-

RNA	Sequence	Chemical formula 5′-P $(M+H)^+$; 5′-NAD $(M)^+$	Mass (m/z) calculated/ found
**RNA-1a**	FurAMP-CAGUAUU	$C_{80}H_{98}N_{29}O_{58}P_8^+$	2640.35/ 2636.04
**RNA-1b**	FurNAD-CAGUAUU	$C_{91}H_{111}N_{31}O_{65}P_9^+$	2956.40/ 2956.13
**RNA-1c**	AMP-CAGUAUU	$C_{80}H_{98}N_{29}O_{58}P_8^+$	2574.34/ 2572.19
**RNA-1d**	NAD-CAGUAUU	$C_{87}H_{109}N_{31}O_{64}P_9^+$	2890.38/ 2890.66
**RNA-2a**	FurAMP-CAUUAUUUGGUAUGCCCGU	$C_{192}H_{237}N_{68}O_{146}P_{20}$	6451.80/ 6455.7
**RNA-2b**	FurNAD-CAUUAUUUGGUAUGCCCGU	$C_{203}H_{250}N_{70}O_{153}P_{21}^+$	6767.85/ 6773.6
**RNA-3a**	FurAMP-R_{Sp}-CAGUAUU	$C_{85}H_{107}N_{29}O_{64}P_9^+$	2836.36/ 2839.63
**RNA-3b**	FurNAD-R_{Sp}-CAGUAUU	$C_{96}H_{120}N_{31}O_{71}P_{10}^+$	3152.41/ 3153.8

Supplementary Table S2. Synthesized dinucleotides, which were incorporated into the 5′-end of different 25mer RNAs using indicated ds 25mer DNA templates. IVT with T7 RNAP in the presence of initiator dinucleotides DN-1a – DN-3a resulted in RNA-5A/G/U

RNA	Sequence	Mass (m/z) calculated/ found
**DN-1a - DN-4a**	^{Fur}AMP-N (N = A (**1a**), G (**2a**), U (**3a**), C (**4a**)	See Figure S7
**DN-1b - DN-4b**	^{Fur}NAD-N (N = A (**1b**), G (**2b**), U (**3b**), C (**4b**)	See Figure S7
**RNA5-A**	(^{Fur}AMP)-A CAG CTC AGC CTA CGA GCC TGA GCC	8425.1/ 8429.3
**RNA5-G**	(^{Fur}AMP)-G ACG CTC AGC CTA CGA GCC TGA GCC	8440.9/ 8447.5
**RNA5-U**	(^{Fur}AMP)-U CAG CTC AGC CTA CGA GCC TGA GCC	not determined
**25mer-A-Templ.**	TAA TAG GAC TCA CTA TTA CAG CTC AGC CTA CGA GCC TGA GCC	
**25mer-G-Templ.**	TAA TAG GAC TCA CTA TAG ACG CTC AGC CTA CGA GCC TGA GCC	
**25mer-U-Templ.**	TAA TAG GAC TCA CTA TTU CAG CTC AGC CTA CGA GCC TGA GCC	

Supplementary Table S3: DNA sequence of cloned enzymes, used in this study.

Enzyme	DNA Sequence (attached restriction sites are underlined)
NudA	CCATGGCGAAAAAGCTGCAAATTGCGGTAGGTATTATTCGCAACGAGAACAAT<br>GAAATCTTTATAACGCGTCGCGCAGCAGATGCGCACATGGCGAATAAACTGGA<br>GTTTCCCGGCGGTAAAATTGAAATGGGTGAAACGCCGGAACAGGCGGTGGTG<br>CGTGAACTTCAGGAAGAAGTCGGGATTACCCCCCAACATTTTTCGCTATTTGAA<br>AAACTGGAATATGAATTCCCGGACAGGCATATAACACTGTGGTTTTGGCTGGT<br>CGAACGCTGGGAAGGGGAGCCGTGGGGTAAAGAAGGGCAACCCGGTGAGTG<br>GATGTCGCTGGTCGGTCTTAATGCCGATGATTTTCCGCCAGCCAATGAACCGGT<br>AATTGCGAAGCTTAAACGTCTGGAAAACCTGTATTTTCAGCTCGAG
NudB	CCATGGAGAAGGATAAAGTGTATAAGCGTCCCGTTTCGATCTTAGTGGTCATCT<br>ACGCACAAGATACGAAACGGGTGCTGATGTTGCAGCGGCGTGACGATCCCGA<br>TTTCTGGCAGTCGGTAACCGGCAGCGTGGAAGAGGGTGAAACCGCGCCGCAA<br>GCTGCCATGCGCGAAGTAAAGGAAGAGGTCACCATTGATGTTGTCGCTGAACA<br>ACTGACCTTAATTGACTGTCAGCGCACGGTAGAGTTTGAAATTTTTTCACATTT<br>ACGTCATCGCTATGCGCCGGGCGTGACGCGTAATACGGAATCATGGTTCTGTCT<br>TGCGCTTCCGCACGAGCGGCAGATCGTTTTCACTGAACATCTGGCTTACAAGTG<br>GCTTGATGCGCCTGCTGCGGCGGCGCTCACTAAGTCCTGGAGCAACCGGCAGG<br>CGATTGAACAGTTTGTAATTAACGCTGCCGAAAACCTGTATTTTCAGCTCGAG
NudC	TCTAGAAATAATTTTGTTTAACTTTAAGAAGGAGATATAATGGATCGTATAATT<br>GAAAAATTAGATCACGGCTGGTGGGTCGTCAGCCATGAACAAAAATTATGGTT<br>GCCGAAGGGAGAATTGCCATATGGCGAAGCGGCAAATTTCGATCTTGTGGGTC<br>AGCGCGCACTACAAATCGGCGAATGGCAGGGGGAACCTGTTTGGTTAGTACAA<br>CAGCAGCGGCGTCACGATATGGGGTCGGTACGTCAGGTCATTGATCTCGATGT<br>TGGGCTGTTTCAACTGGCCGGACGAGGCGTACAACTGGCGGAGTTTTACCGAT<br>CGCATAAATACTGTGGTTACTGCGGGCATGAAATGTATCCGAGCAAAACCGAA<br>TGGGCGATGCTGTGCAGCCATTGCCGTGAGCGTTACTACCCGCAAATCGCCCC<br>CTGCATTATTGTTGCCATCCGTCGCGATGATTCGATCCTCCTCGCCCAGCATAC<br>CCGCCATCGTAACGGTGTCCATACAGTACTTGCCGGATTCGTCGAAGTGGGCG<br>AAACCCTCGAGCAGGCAGTCGCGCGGGAAGTGATGGAAGAGAGCGGAATTAA<br>AGTTAAAAACTTGCGTTACGTGACTTCTCAGCCGTGGCCGTTTCCTCAGTCTTTA<br>ATGACCGCGTTTATGGCGGAATATGACAGCGGCGACATCGTGATCGACCCGAA<br>AGAATTGCTCGAGGCGAACTGGTATCGCTATGACGATTTGCCGTTACTCCCGCC<br>GCCCGGCACCGTAGCGCGCCGTCTGATAGAAGATACGGTGGCGATGTGTCGGG<br>CAGAGTATGAGCTGGTGCCGCGCGGCAGCGCGGCCGCACTCGAG
NudD	CCATGGATTTTTTACGTCAGGAAGACTTTGCCACGGTAGTGCGCTCCACTCCGC<br>TTGTCTCTCTCGACTTTATTGTCGAGAACAGTCGCGGCGAGTTTCTGCTTGGCAA<br>AAGAACCAACCGCCCGGCGCAGGGTTACTGGTTTGTGCCGGGAGGGCGCGTGC<br>AGAAAGACGAAACGCTGGAAGCCGCATTTGAGCGGCTGACGATGGCGGAACT<br>GGGGCTGCGTTTGCCGATAACAGCAGGCCAGTTTTACGGTGTCTGGCAGCACTT<br>TTATGACGATAACTTCTCTGGCACGGATTTCACCACTCACTATGTGGTGCTCGG

	TTTTCGCTTCAGAGTATCGGAAGAAGAGCTGTTACTGCCGGATGAGCAGCATGACGATTACCGCTGGCTGACGTCGGACGCGCTGCTCGCCAGTGATAATGTTCATGCTAACAGCCGCGCCTATTTCTCGCTGAGAAGCGTACCGGAGTACCCGGATTAGAAAACCTGTATTTTCAGCTCGAG
NudE	CCATGGCTAGCAAATCATTACAAAAACCCACCATTCTGAATGTTGAAACTGTAGCCCGTTCCCGACTGTTTACCGTCGAGAGCGTGGATCTGGAGTTCAGCAATGGCGTGCGGCGTGTTTATGAACGAATGCGTCCAACCAACCGGGAAGCAGTGATGATTGTGCCGATTGTGGACGATCACCTGATCCTGATCCGCGAATACGCAGTGGGAACTGAATCCTACGAATTAGGTTTTTCGAAAGGATTAATTGATCCGGGTGAAAGCGTCTACGAAGCCGCTAACCGCGAGCTAAAAGAAGAGGTTGGATTTGGAGCGAACGATCTGACTTTTTTGAAGAAGCTCAGCATGGCACCGTCTTACTTTTCCAGCAAAATGAATATCGTGGTAGCGCAAGATCTCTACCCGGAATCACTGGAAGGCGATGAGCCAGAGCCGCTACCACAGGTGCGCTGGCCGCTGGCGCATATGATGGATTTGCTGGAAGACCCTGACTTCAATGAAGCGCGCAATGTCAGTGCGCTGTTCCTCGTGCGCGAATGGTTGAAAGGGCAGGGGCGAGAAAACCTGTATTTTCAGCTCGAG
NudF	TCTAGAAATAATTTTGTTTAACTTTAAGAAGGAGATATACCATGCTTAAGCCAGACAACCTGCCCGTTACATTTGGCAAAAACGATGTAGAAATTATTGCACGAGAAACACTTTATCGCGGCTTTTTTTCATTAGATCTTTATAGATTTCGTCATCGTCTATTCAACGGGCAAATGAGTCATGAGGTGCGGCGGGAAATTTTTGAGCGCGGTCACGCCGCAGTCTTGCTACCCTTTGACCCAGTGCGTGATGAAGTTGTGCTGATTGAGCAGATTCGGATTGCCGCGTACGACACCAGCGAAACCCCTTGGCTACTGGAGATGGTTGCCGGGATGATTGAAGAGGGTGAAAGTGTGGAAGATGTTGCCCGTCGCGAAGCGATTGAAGAGGCGGGACTGATAGTCAAACGGACCAAACCGGTGTTAAGTTTCCTGGCAAGCCCGGGGGGCACCAGTGAGCGTTCGTCAATTATGGTGGGCGAAGTGGACGCCACGACCGCAAGCGGTATTCACGGTCTGGCTGATGAAAACGAAGATATTCGCGTTCATGTGGTAAGCCGGGAACAGGCATACCAGTGGGTAGAAGAGGGGAAAATCGACAACGCAGCGTCGGTCATCGCTTTGCAATGGCTGCAGCTGCATCATCAAGCGTTAAAAAATGAGTGGGCACTGGTGCCGCGCGGCAGCCTCGAG
NudI	CCATGGATCGACAACGGACTATTGTATGCCCTTTGATTCAAAATGATGGTGCTTATTTGCTGTGTAAAATGGCCGACGATCGCGGCGTTTTCCCCGGTCAATGGGCGATTTCGGGTGGCGGCGTGGAGCCTGGCGAACGAATTGAAGAGGCACTACGCCGCGAAATTCGCGAAGAACTGGGAGAACAGCTGCTTTTGACAGAAATCACGCCGTGGACCTTCAGCGATGATATTCGCACCAAGACGTATGCAGATGGTCGCAAGGAAGAGATTTATATGATTTACCTGATTTTTGACTGCGTTTCTGCCAACCGAGAAGTGAAAATAAACGAAGAGTTTCAGGACTACGCGTGGGTAAAACCTGAAGATCTGGTGCATTATGATTTGAATGTCGCCACCCGAAAAACGTTACGTTTGAAAGGTCTTCTGGAAAACCTGTATTTTCAGCTCGAG
NudJ	CCATGGCGTTTAAACCGCACGTTACCGTTGCTTGCGTGGTGCACGCAGAAGGCAAATTTTTAGTCGTTGAAGAGACGATTAATGGTAAAGCGTTATGGAACCAACCTGCCGGGCATCTGGAAGCCGATGAAACCTTAGTGGAAGCCGCCGCCCGTGAACTGTGGGAAGAAACCGGCATCAGCGCGCAGCCGCAACACTTTATTCGTATGCAT

	CAGTGGATTGCGCCAGATAAAACGCCGTTTTTGCGTTTCCTCTTTGCCATTGAG<br>CTTGAGCAAATATGCCCGACCCAGCCTCATGACAGCGATATCGACTGCTGCCG<br>TTGGGTCAGCGCCGAAGAAATTTTACAGGCGTCAAATCTTCGTTCGCCGCTGGT<br>GGCGGAAAGTATTCGTTGTTATCAAAGCGGGCAACGTTATCCGCTGGAGATGA<br>TTGGCGATTTTAACTGGCCTTTTACAAAGGGTGTCATCGAAAACCTGTATTTTC<br>AGCTCGAG
NudL	CCATGGAATACCGTAGCCTGACGCTTGATGATTTTTTATCGCGCTTTCAACTTTT<br>GCGCCCGCAAATTAACCGGAAACCCTAAATCATCGTCAGGCTGCTGTGTTAAT<br>CCCCATCGTCCGTCGACCGCAACCGGGGTTGTTGCTGACTCAGCGTTCGATTCA<br>TCTGCGTAAACACGCTGGACAAGTGGCATTCCCTGGAGGTGCAGTCGATGACA<br>CGGACGCATCAGCTATCGCCGCCGCGCTGCGCGAAGCTGAAGAAGAGGTCGC<br>TATACCGCCTTCCGCCGTTGAAGTTATCGGCGTGCTGCCGCCCGTCGATAGCGT<br>CACTGGCTACCAGGTAACCCCAGTGGTCGGCATTATCCCGCCCGATCTGCCGT<br>ATCGCGCCAGTGAAGATGAAGTCTCGGCGGTGTTTGAAATGCCGCTCGCCCAG<br>GCATTACATCTGGGTCGTTATCACCCTTTAGATATCTACCGCCGTGGTGATTCA<br>CATCGGGTATGGCTGTCCTGGTACGAACAGTATTTTGTATGGGGAATGACCGC<br>AGGCATAATTCGTGAGCTGGCGCTGCAAATTGGTGTGAAACCCGAAAACCTGT<br>ATTTTCAGCTCGAG
hNudt5	CCATGGAGTCCCAGGAGCCGACCGAGTCGTCGCAGAACGGCAAACAATACAT<br>TATCAGTGAAGAACTGATTAGTGAAGGCAAATGGGTAAAACTGGAAAAAACC<br>ACTTATATGGATCCTACGGGCAAAACGCGCACCTGGGAAAGCGTGAAGCGCA<br>CCACGCGCAAAGAACAGACCGCGGATGGAGTGGCTGTAATCCCAGTGCTGCA<br>GCGTACGTTACATTACGAATGTATTGTGCTTGTCAAGCAGTTCCGCCCGCCTAT<br>GGGAGGATATTGCATTGAATTCCCTGCAGGCTTAATCGATGACGGAGAGACCC<br>CAGAAGCGGCAGCCTTGCGTGAGCTGGAAGAAGAAACAGGTTACAAGGGCGA<br>TATTGCTGAATGTTCACCGGCGGTGTGTATGGACCCAGGTTTGAGTAATTGCAC<br>CATTCATATTGTAACGGTAACCATCAACGGAGATGATGCCGAGAATGCGCGGC<br>CGAAACCGAAACCGGGTGATGGAGAATTCGTCGAAGTTATTTCGCTGCCAAAA<br>AATGATTTGCTGCAGCGCCTGGACGCCCTGGTAGCGGAAGAACATTTAACGGT<br>CGATGCGCGCGTATATTCCTATGCGCTGGCGCTCAAACATGCAAATGCAAAAC<br>CATTTGAAGTCCCTTTTCTGAAGTTTCTGGTGCCGCGCGGCAGCCTCGAG

Höfer, K., Abele, F., Schlotthauer, J., and Jäschke, A. (2016). Synthesis of 5'-NAD-Capped RNA. Bioconjug Chem *27*, 874-877.
Liu, R., and Visscher, J. (1994). A novel preparation of nicotinamide mononucleotide. Nucleosides Nucleotides *13*, 1215-1216.
